# Reduced incidence of respiratory, gastrointestinal and malaria infections among children during the COVID-19 pandemic in Western Kenya: An analysis of facility-based and weekly diaries data

**DOI:** 10.7189/jogh.13.06024

**Published:** 2023-07-14

**Authors:** Gloria P Gómez-Pérez, Richard de Groot, Amanuel A Abajobir, Caroline W Wainaina, Tobias F Rinke de Wit, Estelle Sidze, Menno Pradhan, Wendy Janssens

**Affiliations:** 1 Amsterdam Institute for Global Health and Development, Amsterdam, the Netherlands; 2 PharmAccess Foundation, Amsterdam, the Netherlands; 3 African Population and Health Research Centre, Nairobi, Kenya; 4 Universiteit Utrecht, Amsterdam, the Netherlands; 5 Vrije Universiteit, Amsterdam, the Netherlands; 6 Universiteit van Amsterdam, the Netherlands

## Abstract

**Background:**

Epidemics can cause significant disruptions of essential health care services. This was evident in West-Africa during the 2014-2016 Ebola outbreak, raising concerns that COVID-19 would have similar devastating consequences for the continent. Indeed, official facility-based records show a reduction in health care visits after the onset of COVID-19 in Kenya. Our question is whether this observed reduction was caused by lower access to health care or by reduced incidence of communicable diseases resulting from reduced mobility and social contacts.

**Methods:**

We analysed monthly facility-based data from 2018 to 2020, and weekly health diaries data digitally collected by trained fieldworkers between February and November 2020 from 342 households, including 1974 individuals, in Kisumu and Kakamega Counties, Kenya. Diaries data was collected as part of an ongoing longitudinal study of a digital health insurance scheme (Kakamega), and universal health coverage implementation (Kisumu). We assessed the weekly incidence of self-reported medical symptoms, formal and informal health-seeking behaviour, and foregone care in the diaries and compared it with facility-based records. Linear probability regressions with household fixed-effects were performed to compare the weekly incidence of health outcomes before and after COVID-19.

**Results:**

Facility-based data showed a decrease in health care utilization for respiratory infections, enteric illnesses, and malaria, after start of COVID-19 measures in Kenya in March 2020. The weekly diaries confirmed this decrease in respiratory and enteric symptoms, and malaria / fever, mainly in the paediatric population. In terms of health care seeking behaviour, our diaries data find a temporary shift in consultations from health care centres to pharmacists / chemists / medicine vendors for a few weeks during the pandemic, but no increase in foregone care. According to the diaries, for adults the incidence of communicable diseases/symptoms rebounded after COVID-19 mobility restrictions were lifted, while for children the effects persisted.

**Conclusions:**

COVID-19-related containment measures in Western Kenya were accompanied by a decline in respiratory infections, enteric illnesses, and malaria / fever mainly in children. Data from a population-based survey and facility-based records aligned regarding this finding despite the temporary shift to non-facility-based consultations and confirmed that the drop in utilization of health care services was not due to decreased accessibility, but rather to a lower incidence of these infections.

Previous epidemics in Sub-Saharan Africa, like the outbreak of Ebola in West-Africa in 2014-2016, had devastating consequences for the provision of essential health care services, resulting in thousands of indirect crisis-related deaths [1-4]. In Sierra Leone, there was a significant decrease in utilization of services for antenatal and postnatal care, associated with marked increases in maternal mortality ratio and stillbirth [5]. In malaria-endemic countries like Guinea, Liberia, and Sierra Leona, the emergence of Ebola constituted a heavy blow to malaria control efforts. In Guinea alone, 74 000 fewer malaria cases were seen as compared to the years without Ebola [6]. Most alarmingly, there were an additional 7000 malaria-related deaths in children younger than five years old in Ebola-affected countries. Human immunodeficiency virus (HIV) / acquired immunodeficiency syndrome (AIDS)- and tuberculosis-related deaths also increased significantly during the Ebola outbreak [7]. Therefore, there were great concerns at the onset of the COVID-19 pandemic about similar impacts on the supply and utilization of non-COVID-19-related health care services in Africa.

Evidence from low- and middle-income countries (LMICs) shows that the COVID-19 pandemic has resulted in a reduced utilization of health care services for a wide range of essential curative care as well as antenatal and postnatal care, institutional deliveries, and immunization [8-17]. In many countries this reduced demand is thought to be driven by fears of patients (and / or health care staff) of contracting COVID-19 [18,19]. Poverty could also cause reduced demand for health care, exacerbated by massive loss of African jobs due to COVID-19 [20,21] and the fact that most health care in Africa is paid out-of-pocket [22-27]. Restrictions in mobility due to curfews and lockdowns and reduced availability of transport could be other obstacles to access health care facilities.

The COVID-19 pandemic also affects the supply-side of the health care system. Healthcare facilities might become overburdened with high numbers of COVID-19 patients, including time- and resource-intensive triage requirements [28,29]. Staff may be absent due to illness or travel restrictions [30], or unwilling to work out of fear of infection when personal protection equipment (PPE) is in short supply [13,31]. Additionally, worldwide supply chains of medicines, PPEs, and diagnostic supplies have been faltering due to the global lockdowns [32,33].

Whereas the decreased offer of health services in industrialized countries is partially a result of facilities’ restrictions for non-COVID-19 patients, in LMICs it is mainly owed to the lack of epidemic preparedness and minimal resilience of health care systems [34]. For instance, compared to 2019, Global Fund-supported HIV, tuberculosis and malaria services dropped significantly in Africa and Asia in 2020 [35]. Moreover, the current pandemic has also negatively affected the routine care of non-communicable diseases (NCDs) in Sub-Saharan Africa [10].

Importantly, non-pharmaceutical COVID-19 interventions, including lockdowns, school closures, and curfews, have collaterally impacted the seasonal prevalence of other viral and bacterial infections with aerosol transmission [36,37]. For instance, several industrialized countries have experienced a much less severe flu season during the COVID-19 pandemic compared to previous years [37-44], and a lower prevalence of enteric viral infections [45-49]. A study across six continents reported a declined prevalence of invasive bacterial infections due to organisms typically transmitted via respiratory droplets [50]. Likewise, on the African continent, South-Africa has observed a lower prevalence of flu-like disease and respiratory syncytial virus detection during the pandemic [15,51]. Further evidence from Sub-Saharan Africa on the COVID-19 effects on communicable diseases is mostly lacking.

The above led to the hypothesis that both the incidence of health care events and health care utilization conditional on being ill would decrease.

In LMICs as in high-income countries, surveillance systems are of great importance to provide information regarding underlying trends in health conditions and health care utilization, also in the context of COVID-19 and its containment measures. Importantly, such surveillance systems should include both facility- and community-based sources of information. Crucially, the former cannot distinguish between reduced demand due to lower incidence or to decreased access (financial, geographical, mental, supply-side barriers), nor shed light on shifts from formal to informal care versus foregone care. In contrast, community-based data can investigate these patterns and triangulate facility-based data.

In this paper we study changes in the need for and utilization of health care services in Western Kenya associated with the outbreak of the COVID-19 pandemic. We use highly detailed high-frequency, cohort-based ‘Diaries’ data, as well as official facility-based data, from a rural population in Western Kenya throughout a pre- and post-COVID-19 period, to examine direct and indirect impacts of the COVID-19 pandemic on incidence / prevalence of health symptoms and on health care utilization. The almost year-long, weekly diaries data allow us to measure health-seeking behaviour for both minor and major health symptoms with great accuracy and limited recall bias [52]. For instance, health diaries have been successfully used as community-based surveillance tools in rural Kenya to study the transmission dynamics of respiratory viruses [53], as well as to analyse risk-coping mechanisms of households with and without health insurance [54]. We assess the impacts of COVID-19 separately for adults and children. We also explore to what extent the timing of COVID-19 containment measures was associated with the incidence of self- and facility-reported health events and health care utilization.

## METHODS

### Study design and participants

We performed a quantitative prospective cohort study with baseline and endline surveys collected in October / November 2019 and November 2020, and weekly diaries interviews from February 2020 to November 2020, in Kakamega and Kisumu Counties. We triangulated these data with monthly administrative data from all health facilities in both counties, covering the same period as the diaries in 2020, as well as the two preceding years to investigate independent seasonal trends. The counties are located in the West of Kenya, close to Lake Victoria and the Ugandan border. Kakamega County boasts a population of 1.9 million people and Kisumu has 1.2 million inhabitants [55]. Except for Kisumu town, the counties are predominantly rural with just under half (49%) of all households in Kisumu and 77% of households in Kakamega engaged in agriculture [56]. The health care sector mainly consists of public providers, private practices, and faith-based organizations, with public facilities dominating the sector [57,58].

### Data sets

We use two primary data sources. The first data set comes from the Financial and Health Diaries study (henceforth Diaries), originally conceptualized to evaluate the impact of a mobile-phone based health insurance scheme in Kakamega County, and the implementation of universal health coverage in Kisumu County (Trial registries: AEA Registry [AEARCTR-0006089] and ClinicalTrials.gov [NCT04068571]) [59]. Both schemes were on hold in our study area during most of the first year of the COVID-19 pandemic. Nevertheless, some of the study households in Kakamega County were offered digital health insurance from July 2020 onwards, and by November 2020 a third of the households here studied had access to health insurance. The Diaries data cover the weeks between February 4-November 23, 2020. The study participants were recruited from low-income rural villages and consisted of a representative sample of households complying with the following eligibility criteria: having at least one woman aged 18-49 years old, who was either pregnant or with a child under four years old at the time of the baseline. The sampling methodology was based on a stratified two-stage random sampling strategy, with stratification along two counties (Kisumu and Kakamega). Within each county, health facilities were purposely selected, two in Kisumu and four in Kakamega. Next, we randomly selected catchment areas around each health facility, four per facility in Kisumu and six per facility in Kakamega. Finally, we randomly selected ten households per catchment area from a list of all eligible households within them. More details on the sample selection and research methodology have been published elsewhere [59].

The Diaries sample consists of 239 households in Kakamega and 103 households in Kisumu, which were first interviewed for a baseline survey during October-November 2019, encompassing 1974 household members. In December 2019, the weekly Diaries data collection started with 630 adult respondents (414 women and 216 men). December 2019 and January 2020 data are excluded from the analysis due to incomplete data collection during Christmas. Each week, respondents were asked about any health events in their household (i.e. occurring to themselves or other household members, including children) and health events were recorded for each household member separately. If a health event occurred, follow-up questions were asked about the type of health event, health care-seeking, services received and expenditures. We use data on the (self-reported) occurrence of minor and major illnesses, symptoms, health care utilization, provider type, and foregone care. After the first case of COVID-19 was registered in Kenya in March 2020, and the government implemented mobility restrictions, the field work switched from in-person interviewing to phone interviews. Due to the strong built-up rapport and trust between field workers and respondents during the three pre-COVID months, this transition occurred with minimal disruptions (Figure S1 in the **Online Supplementary Document**).

The second data set comes from the District Health Information System of Kenya (DHIS2). We obtained monthly data from the DHIS2 for the years 2018, 2019 and 2020, aggregated at the county level. These data have been widely used to assess population-level health outcomes and health care utilization, despite some implementation challenges for data entry [60,61]. The DHIS2 data includes the number of cases reported for 90 categories of illnesses or health problems (Table S1 in the **Online Supplementary Document**). We conducted the same categorization process for the Diaries data using a list of 47 common health symptoms (Table S2 in the **Online Supplementary Document**).

Description of the COVID-19 epidemiology and containment measures in the study area.

**Figure 1:**
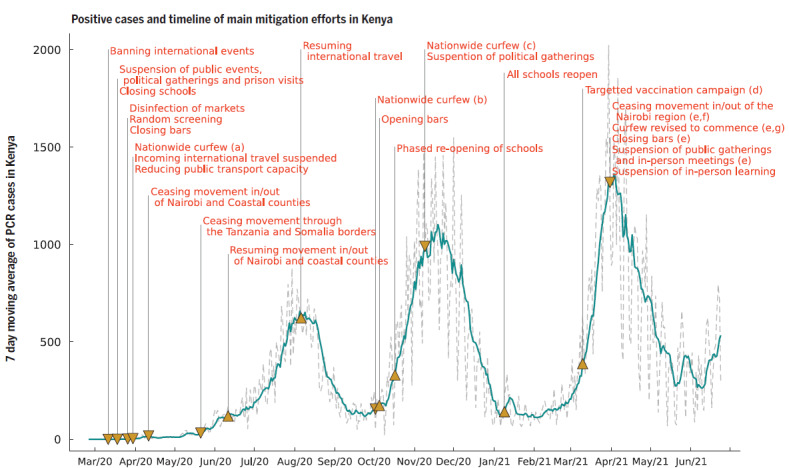
Seven-day moving average of daily positive PCR tests from the Kenyan national linelist and a timeline of the main mitigation events applied by the Kenyan government. Figure extracted from Brand et al, 2021 [62]. Down-arrow represents tightening of measures, and up-arrows relaxation of measures. (a) Curfew from 7 pm to 5 am; (b) curfew from 11 pm to 4 am; (c) curfew from 10 pm to 4 am; (d) front-line workers and individuals older than 58 years ( ~ 1.2 million doses); (e) the region includes Nairobi, Kajiado, Machakos, Kiambu, and Nakuru; (f) this restricted movement into and out of the block of counties in (e) but not between these counties; (g) curfew from 8 pm to 4 am.

The COVID-19 pandemic officially arrived in Kenya on March 13, 2020, and the government immediately implemented measures to contain the spread of the virus including: closure of schools, bars, and restaurants; a ban on social gatherings; working-from-home directions; dawn-to-dusk curfews; and closure of international borders (**Figure 1**) [62]. The nationwide curfew installed on March 27, 2020 included ceasing of movement in / out of the most affected areas, including Nairobi, Mombasa, Kilifi, and Kwale, and through the Tanzania and Somalia borders [63]. In 2020, these travel restrictions were lifted in June and July; national flights resumed mid-July, with international flights resuming early August. The dawn-to-dusk curfew and closure of schools remained in place until October 2020. As of July 27, 2020 (around the peak of the first COVID-19 wave in Kenya) (**Figure 1**), there were nearly 18 000 confirmed cases in Kenya and 285 deaths on a population of almost 50 million. The second wave had a greater impact and started in October 2020. According to official records, our study areas were affected with 152 COVID-19 cases in Kisumu between June and October 2020 [64], and 316 in Kakamega at the end of October 2020 [65]. However, due to the frequent asymptomatic / mildly symptomatic COVID-19 clinical presentation in Africa, many individuals did not get tested. It has been estimated that in the WHO Africa region only 1.4% (one in 71) of SARS-CoV-2 infections are reported [66]. Hence, the actual COVID-19 cases in this community are significantly larger.

### Outcome variables

Our primary individual-level outcome variables in the Diaries data included incidence (acute diseases) or prevalence (chronic conditions) of a health problem in the past seven days (measured with a dummy variable), healthcare utilization conditional on experiencing a health problem in the past seven days (measured with a dummy variable), and choice of healthcare provider: formal (doctor, nurse, or midwife), informal (traditional healer / herbalist, community health volunteer, or religious person) or pharmacy (pharmacist / chemist / medicine vendor). Health problems were self-reported by the adult participants in the Diaries (or by another knowledgeable household member in case of absence), and by a knowledgeable adult (mostly the mother) for the children. The 47 health symptoms (Table S2 in the **Online Supplementary Document**) were further categorized and aggregated by health condition: gastrointestinal diseases, malaria / fever, respiratory / airborne infections, and NCDs. Illnesses not falling into one of these categories are not reported in this paper. For those who reported a health problem, indicators were constructed whether they sought any consultation (yes / no) and if so, whether they visited a formal or informal provider or a pharmacy. For the interpretation of the results, the pharmacy category was hence not considered informal care, but another type of formal care.

### Ethical approval

The Diaries study was approved by the Amref Health Africa Ethics and Scientific Review Committee (P679-2019, August 8, 2019), with an amendment approved for the additional survey modules and switch to telephone interviews on April 21, 2020. Participants were asked for written informed consent before enrolment in the baseline survey, and separate written consent to participate in the weekly Diaries data collection. Trained fieldworkers administered the consent forms to all adults in the household, as well as to girls younger than 18 years who were pregnant or with a child, as they qualify as emancipated minors (able to give their own consent) according to Kenyan ethical guidelines. The head of the household was asked for consent to collect data from un-emancipated minors prior to the start of the Diaries interviews. The most informed adult household member responded on their behalf.

### Statistical analysis

For the Diaries data, our unit of analysis was the individual by week. The analysis included all individual-weeks with full health information. We observed 1974 unique individuals (785 adults and 1189 children below age 18) in 342 households with at least one observation over the 40-week study period. Our total sample size for the analysis was 55 923 individual-week observations (21 204 for adults and 34 719 for children). We conducted our analysis for the full sample, and for children and adults separately.

We first calculated descriptive baseline characteristics of the sample population, using frequencies and percentages for binary and categorical variables, and means plus standard deviations (SD) for continuous variables. We examined the impact of the COVID-19 pandemic on selected health outcomes by estimating linear probability regressions with household fixed-effects while clustering the standard errors at the household level. We also performed logit regressions and individual fixed-effects analysis, which yielded similar results. Tables with the results of logit regressions can be found in the **Online Supplementary Document**. We estimate weekly changes compared to the pre-COVID mean of the dependent variable (*t* = 0) using the following equation:



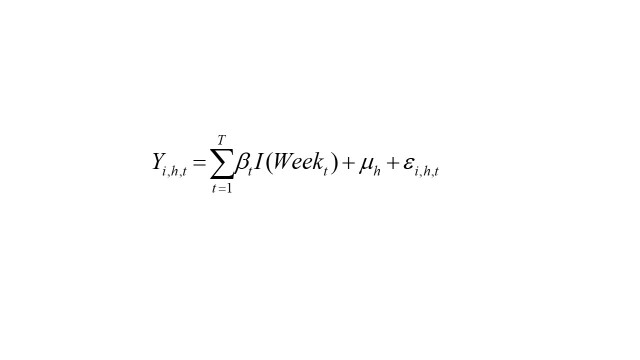

(1)


In equation 1, Y_i,h,t_ indicates the outcome value for dependent variable Y, for individual i in household h during week t; I(Week_t_) represents a dummy indicator for each post-COVID week t, with the pre-COVID weeks all indicated by *t* = 0; μ_h_ are the household fixed-effects and ε_i,h,t_ is a random error term. We were interested in coefficient β_t_, which measures the difference between week t and the pre-COVID average. The pre-COVID mean is calculated by averaging the outcome variable over the weeks from February 4 to March 16, 2020. We present the results from this analysis in graphical form by plotting the point estimates and 95 percent confidence intervals for each week.

For the DHIS2 data, we prepared graphical outputs by showing the number of cases for each illness category by month, comparing across the three years. Due to the nature of this data (simple counts of reported cases), we were not able to conduct additional statistical analysis.

All analyses were conducted using Stata 16.1, including the generation of tables and preparation of figures.

## RESULTS

The average number of observations per individual over the 40-week study period was 37 with a median of 40 observations. The average response rate was 95.6% for our pre-COVID period. A positive response is counted if at least one adult in the household was successfully interviewed, and able to complete the health module for all household members. The response rate dropped slightly after the switch to phone interviewing, but as of May, the response rate picked up to reach pre-COVID levels and surpassed the pre-COVID response rate during the rest of the year (Figure S1 in the **Online Supplementary Document**).

The baseline characteristics of the households included in the study are summarized in **Table 1**. 70% of the sample is from Kakamega; the other 30% from Kisumu. Households have on average five members with three members under 18 years. Household heads (HHHs) are 37 years old on average and 24% are female. Nearly all HHHs (92%) are married. The majority have received some basic education; 31% moved beyond primary school level. The most common occupations of the head are casual labour (39%), operating an informal business (22%) or formal employment (19%). During the 12 months preceding the baseline survey, household members used inpatient formal care on average 3 times, and outpatient formal care nearly 15 times. Pharmacists were also consulted 15 times on average during this period. Traditional healers were consulted less often, on average twice per year, and foregone care was reported to occur on average 3 times per year. Households spent just over 7100 Kenyan Shillings (KES) annually out-of-pocket on health care, equivalent to approximately US dollars (US$) 70 (1 US$ = 101.95 KES at the time of the baseline survey on November 15, 2019).

**Table 1:** Descriptive statistics of the diaries sample population

Characteristics at baseline	Frequency or mean		Percentage or SD
Total households	342	-	
Households per County (frequency, %)			
*Kakamega*	239	69.9%	
*Kisumu*	103	30.1%	
Household composition (mean, SD)			
Household members	5.0	(1.8)	
*Members age 0-5 years*	1.4	(0.7)	
*Members age 6-12 years of age*	1.1	(1.0)	
*Members age 13-18 years of age*	0.5	(0.8)	
*Members age 19-64 years of age*	2.0	(0.7)	
*Members age 65 and over*	0.0	(0.2)	
**Household head characteristics**			
Age in years (mean, SD)	37.1	(10.6)	
Sex (frequency, %)			
*Female*	83	24.3%	
*Male*	259	75.7%	
Marital status (frequency, %)			
*Married*	313	91.5%	
*Not married*	29	8.5%	
Education (frequency, %)			
*No schooling*	12	3.5%	
*Incomplete primary*	110	32.2%	
*Complete primary*	110	32.2%	
*Incomplete secondary*	31	9.1%	
*Complete secondary or higher*	79	22.0%	
Occupation (frequency, %)			
*None*	49	14.4%	
*Own business*	75	22.0%	
*Farm owner*	20	5.9%	
*Casual labor (including casual farm work)*	132	38.7%	
*Wage work*	65	19.1%	
Health care utilization in past 12 months (mean, SD)			
*Number of times inpatient formal care*	3.0	(12.6)	
*Number of times outpatient formal care*	14.5	(12.3)	
*Number of times informal drug vendor*	14.9	(18.6)	
*Number of times traditional healer*	2.0	(5.8)	
*Number of times foregone care*	3.3	(5.5)	
Total health expenditures (KES)	7054	(16 803)	

SD – standard deviation, KES – Kenyan shillings

Before the COVID-19 pandemic started in Kenya, the average weekly prevalence of self-reported health problems in the full sample was 12.2% (Table S3 in the **Online Supplementary Document**). This prevalence dropped significantly with up to 5.9 percentage points after the first COVID-19 case was detected in Kenya for five consecutive weeks between March 24 and April 27, 2020. After this initial period, the prevalence of any health problems in children continued to be lower than before COVID-19 for the rest of the year, while the prevalence among adults moved back to or surpassed pre-COVID levels, except for one week in September and one in October in 2020 (the period between the first and second COVID-19 week when registered COVID-infections were also at a low) (**Figure 1**).

**Figure 2:**
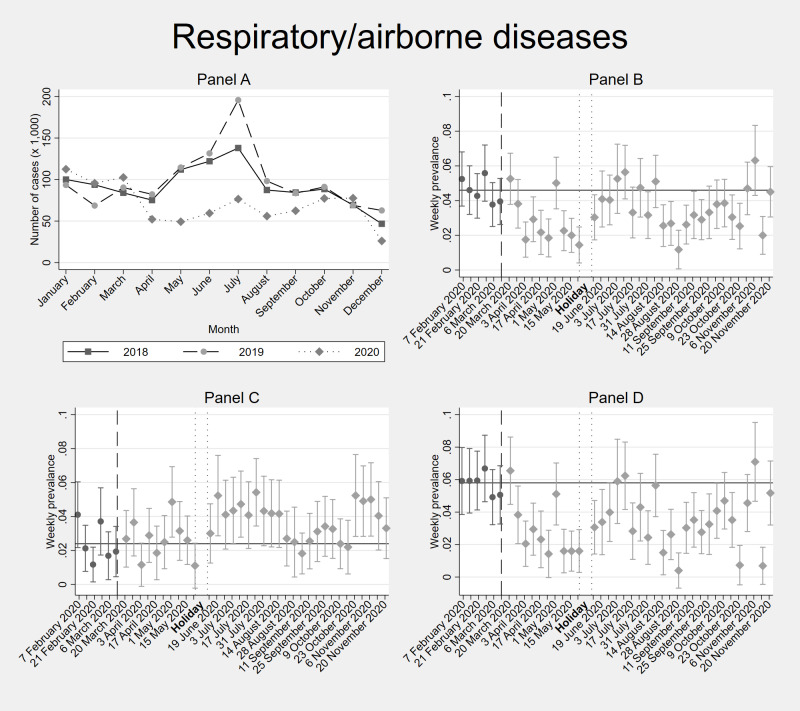
Facility-based and weekly diaries’ incidence data of respiratory / airborne infections in Kisumu and Kakamega counties (Kenya) before and during the COVID-19 pandemic. **Panel A**. shows facility-based data (DHIS2) from the years 2018, 2019 and 2020. **Panel B**. shows 40-weeks weekly diaries data February-November 2020 from all household members. **Panel C**. shows separated incidence data for adults (≥18 years old) within the households. **Panel D**. shows separated incidence data for children (<18 years old) within the households. In **Panel B**, **Panel C** and **Panel D**, the vertical dashed line shows the time cut-off for before and during COVID-19 comparisons; the two dotted vertical lines show the holiday period without interviews; and the horizontal line shows the average incidence / prevalence before COVID-19. Error bars show 95% confidence intervals. DHIS2 – District Health Information Software 2

**Figure 3:**
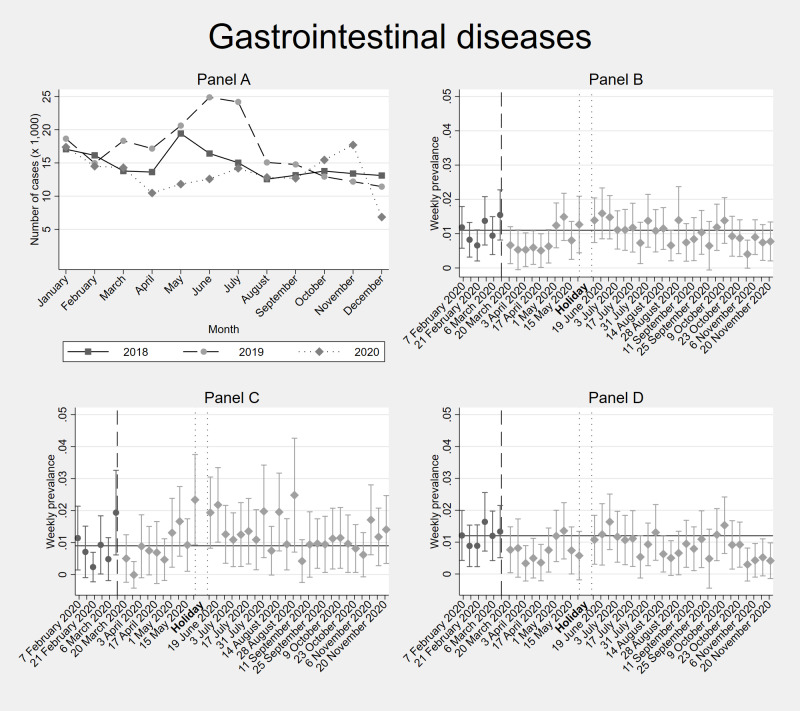
Facility-based and weekly diaries’ incidence data of gastrointestinal diseases in Kisumu and Kakamega counties (Kenya) before and during the COVID-19 pandemic. **Panel A**. shows facility-based data (DHIS2) from the years 2018, 2019 and 2020. **Panel B**. shows 40-weeks weekly diaries data from February-November 2020 from all household members. **Panel C**. shows separated incidence data for adults (≥18 years old) within the households. **Panel D**. shows separated incidence data for children (<18 years old) within the households. In **Panel B**, **Panel C** and **Panel D**, the vertical dashed line shows the time cut-off for before and during COVID-19 comparisons; the two dotted vertical lines show the holiday period without interviews; and the horizontal line shows the average incidence / prevalence before COVID-19. Error bars show 95% confidence intervals. DHIS2 – District Health Information Software 2

**Figure 4:**
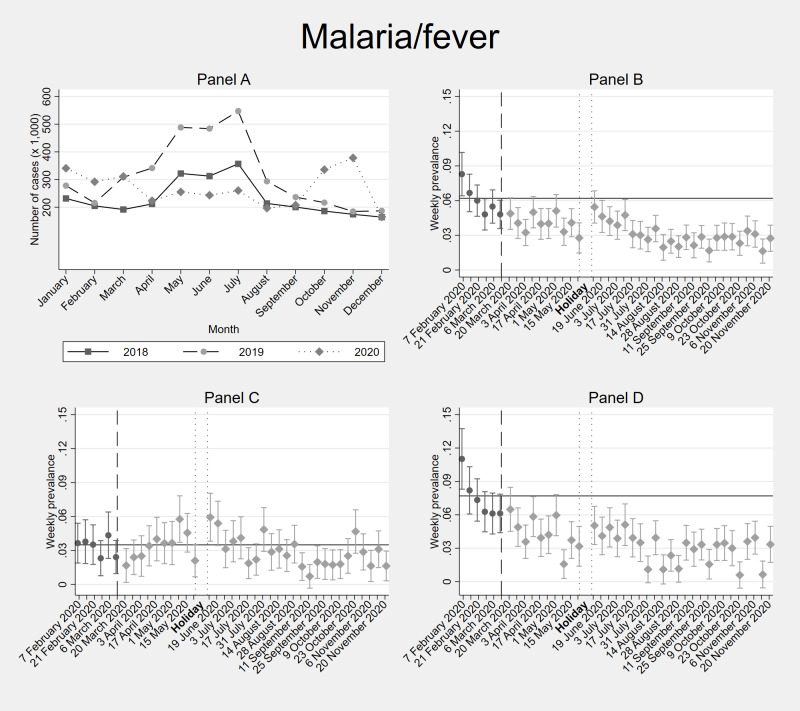
Facility-based and weekly diaries’ incidence data of malaria or fever in Kisumu and Kakamega counties (Kenya) before and during the COVID-19 pandemic. **Panel A.** shows facility-based data (DHIS2) from the years 2018, 2019 and 2020. **Panel B.** shows 40-weeks weekly diaries data from February-November 2020 from all household members. **Panel C**. shows separated incidence data for adults (≥18 years old) within households. **Panel D**. shows separated incidence data for children (<18 years old) within the households. In **Panel B**, **Panel C** and **Panel D**, the vertical dashed line shows the time cut-off for before and during COVID-19 comparisons; the two dotted vertical lines show the holiday period without interviews; and the horizontal line shows the average incidence / prevalence before COVID-19. Error bars show 95% confidence intervals. DHIS2 – District Health Information Software 2

**Figure 5:**
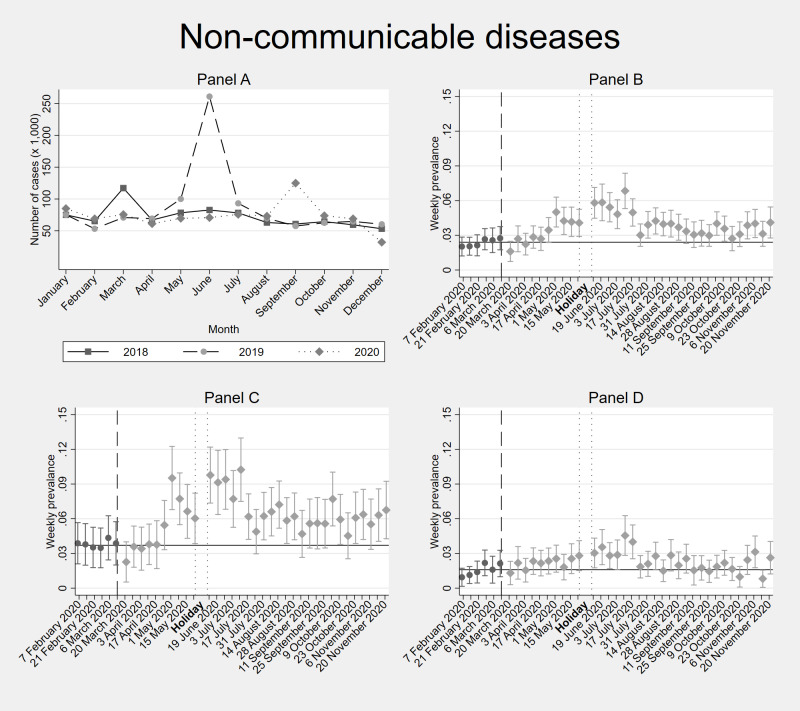
Facility-based and weekly diaries’ incidence data of NCDs diseases in Kisumu and Kakamega counties (Kenya) before and during the COVID-19 pandemic. **Panel A.** shows facility-based data (DHIS2) from the years 2018, 2019 and 2020. **Panel B.** shows 40-weeks weekly diaries data from February-November 2020 from all household members. **Panel C.** shows separated incidence data for adults (≥18 years old) within the households. **Panel D.** shows separated incidence data for children (<18 years old) within the households. In **Panel B**, **Panel C** and **Panel D**, the vertical dashed line shows the time cut-off for before and during COVID-19 comparisons; the two dotted vertical lines show the holiday period without interviews; and the horizontal line shows the average incidence / prevalence before COVID-19. Error bars show 95% confidence intervals. DHIS2 – District Health Information Software 2

**Figure 2**, **Figure 3**, **Figure 4** and **Figure 5** depict the trends in the incidence of recorded health problems for respiratory diseases, gastrointestinal illnesses, malaria / fever, and the prevalence of NCDs, respectively (Tables S4-7 in the **Online Supplementary Document**). Each figure contains four panels: panel A. DHIS – all individuals, panel B. Diaries – all household members, panel C. Diaries – adults only, panel D. Diaries – children only. Panel A shows diagnosed conditions (conditional on seeking care at a formal facility). Panels B, C, and D show self-reported conditions (regardless of whether this resulted in a health care consultation).

The trend for the full Diaries sample in panel B masks the heterogeneity by age. Before COVID-19, the average weekly incidence of self-reported respiratory / airborne infections was 4.6% in the full sample, with 2.5% for adults vs 5.7% for children (**Figure 2**, Table S4 in the **Online Supplementary Document**). After the onset of COVID-19, there was a significant drop in the incidence of these conditions for children. Incidence remained significantly lower for them during almost every week, even during most of the first and second COVID-19 waves, until the end of November, 2020. The adults, on the other hand, reported an incidence of respiratory infections similar to the pre-COVID-19 period, except for two drops in the early stages of the pandemic, and a significantly higher prevalence than before COVID-19 coinciding with the start of both the first (July-August) and second (October-November) COVID-19 waves in 2020.

The weekly post-COVID incidence of self-reported gastrointestinal infections (**Figure 3**, Table S5 in the **Online Supplementary Document**) was significantly lower than before COVID-19 in children during the early stages of the pandemic as well as some weeks in July, August, and November of 2020. For adults instead, pre-post-COVID differences were not significant except for a higher incidence rate in May and June, 2020.

The incidence of weekly self-reported malaria/fever symptoms before COVID-19 was 7.5% for children and 3.3% for the adults (**Figure 4**, Table S6 in the **Online Supplementary Document**). After mid-March 2020, there was a significantly lower incidence of malaria / fever symptoms for children throughout the study period. Adults, too, showed significantly lower incidence in several weeks compared to before COVID-19, but also some weeks during which the incidence of self-reported malaria / fever was higher – especially during the rainy season in May and June 2020.

In contrast, the number of weekly self-reported NCDs increased as compared with the pre-COVID-19 period for both children and adults, but particularly for adults from May onwards (**Figure 5**, Table S7 in the **Online Supplementary Document**). The top five reported NCDs in the diaries were headaches, skin problems / allergy / itching, general body weakness, dental / mouth problems, and backache.

Comparing the diaries with the facility-level DHIS2 data (**Figure 2**, **Figure 3**, **Figure 4** and **Figure 5**), we find that the 2020 trends of health care utilization for respiratory and gastrointestinal infections as observed in the DHIS2 are very similar to the self-reported incidence in the diaries. The same applies for malaria, until before the September-November 2020 period, during which DHIS2 reports an increased malaria incidence compared to 2018 and 2019, while we do not observe this peak of malaria/fever symptoms in the Diaries between September-November 2020. DHIS2 data show an increase in health care utilization for NCDs only in September 2020.

**Figure 6:**
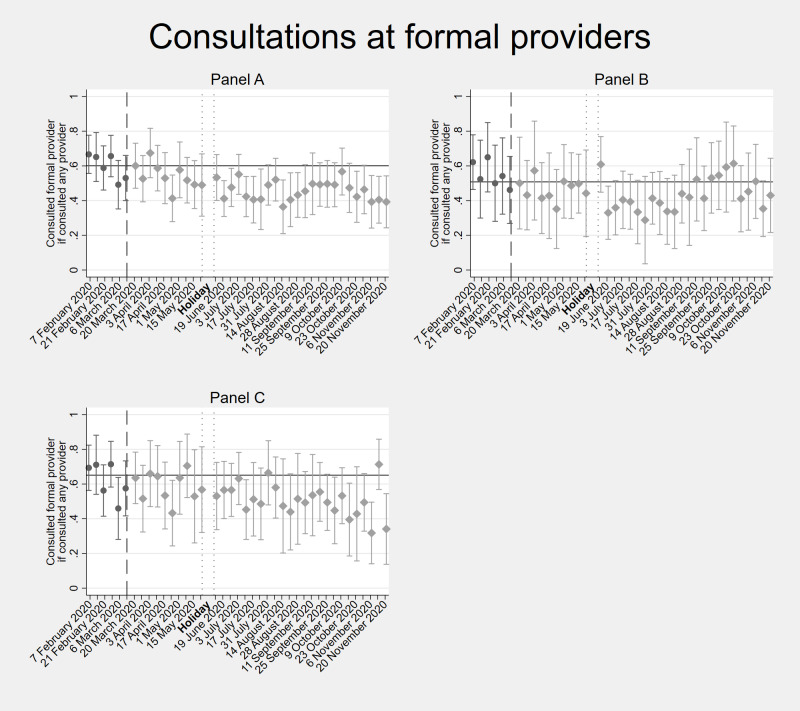
Consultations at formal providers conditional on seeking care. It shows the weekly average number of times that individuals living in these households consulted a formal provider (doctor, nurse, or midwife) at a health care centre conditional on seeking care from February-November 2020. **Panel A.** all household members. **Panel B.** adults (≥18 years old). **Panel C.** children (<18 years old). The vertical dashed line shows the time cut-off for before and during COVID-19 comparisons; the two dotted vertical lines show the holiday period without interviews; and the horizontal line shows the average of consultations before COVID-19. Error bars show 95% confidence intervals.

The Diaries data allow us to directly assess whether individuals who experienced a health problem did or did not seek care at a health care provider. To this end, we analysed visits to formal health care providers for those weeks in which an individual experienced a health problem (i.e. “conditional” consultations). **Figure 6** and Table S8 in the **Online Supplementary Document** show the percentage of individuals consulting a formal provider conditional on seeking care. Before COVID-19, the average rate of conditional care-seeking was 61%. After the onset of COVID-19, this share remained relatively stable for the full sample except for nine out of 34 weeks coinciding with the peaks of the first and second COVID-19 wave. We observed significant declines in conditional consultations among children early May, and during the months of July, August, September (first COVID-19 wave), as well as October and November (second COVID-19 wave) of 2020. For adults, no statistically significant reductions of seeking care were observed, except during some weeks in June and November 2020 (second COVID-19 wave). The shift away from formal health facilities was accompanied by a shift towards consultations at pharmacists and medicine vendors (Figure S2 in the **Online Supplementary Document**), but not to informal providers such as traditional healers.

## DISCUSSION

The COVID-19 pandemic has changed dramatically the dynamics of community social interactions and disrupted the functioning of health care systems worldwide. Containment measures, including lockdowns, curfews, school closures, travel restrictions, physical distancing, use of face-masks, improved compliance with hand-washing, and ventilation, have been guided by our current scientific knowledge about how to prevent the spread of airborne pathogens [67,68] and aim at slowing down the transmission of SARS-CoV-2 to avoid overwhelming the health care system and concomitant high death tolls.

In industrialized countries, the strict COVID-19 containment measures resulted in an unintended but beneficial collateral effect on the epidemiology and seasonality of other infectious diseases transmitted by aerosols/respiratory droplets [36-44,50,51], and by faecal-oral route and fomites, like enteroviruses [46-49], resulting in milder flu seasons and less intestinal infections reported worldwide during the pandemic. Importantly, these epidemiological observations are based on facility data and molecular studies that in industrialized countries likely represent real epidemiological changes, while in LMICs it remains unclear if such observations are indeed a consequence of the non-pharmaceutical interventions and the resulting lower transmission of these infections, or due to disruptions in health care utilization [69]. In Sub-Saharan Africa such disruptions could be caused by fear of contracting COVID-19, similarly to what occurred during previous epidemics in the continent [2,5]; financial barriers due to pandemic-related job insecurity and income loss, that hit hard the economies of many low-income families [21,63,70,71]; travel restrictions; and the fragility and lack of epidemic preparedness of the health care systems impeding the continuity of essential health care services [28,34,70]; among other reasons.

To disentangle the above causalities, we compared facility-based DHIS2-data with weekly diaries data in Kisumu and Kakamega. Importantly, the highly granular weekly data of the diaries allowed us to make the distinction between an observed reduction in health care utilization due to reduced access, and reduced utilization due to an actual reduction in incidence or prevalence. Given that the weekly interviews were initiated before COVID-19 arrived in Kenya, and continued throughout the pandemic with minimal disruptions, the study was able to detect a break in typical trends due to COVID-19.

We provide evidence that the radical reduction of social contacts and stringent mobility restrictions during the pandemic, affected temporarily the health-seeking behaviour of the population (shifting from formal facilities to pharmacist / drug vendors), mostly during the two first COVID-19 waves in Kenya; and changed the epidemiology and seasonality of some infections and NCDs.

We observed a significant reduction of respiratory infections, most strongly among children. This stresses the important role that restriction of social contacts (schools, gatherings, bars, restaurants, places of worship) had on the transmission of respiratory infectious agents in Kenya, were a reduction of 62% of physical contacts have been estimated during the pandemic in informal settings [72]. Even before COVID-19, evidence suggested that school closures were associated with reduced incidence of influenza, and that this effect was the greatest among school-aged children [73]. Since school closures were in place from March 2020-January 2021 (except for a short period in November 2020 during final exams), while other measures were on and off through the year 2020, this could also explain the fact that the reduction was most significant among children. As in most low income countries [74], adults could not afford to stay at home completely, since they needed to secure an income. In some urban slums, employment and job hunting were among the main reasons for adults to use public transportation, and to travel outside their communities during the pandemic [75]. Moreover, adults in the poorest quintile in informal Kenyan settlements reported 1.5 times more social contacts that those in the richest [72]. The weeks during which adults in our diaries reported a higher incidence of respiratory infections (**Figure 2**), overlap with the two COVID-19 waves (**Figure 1**). Since in Africa the rate of asymptomatic / sub-clinical COVID-19 infections was very high [76-79], it is well possible that many of these respiratory infections were mild COVID-19 cases. This highlights the vulnerability of low-income populations in times of epidemics, and the difficulties they face to follow certain control measures.

The modest impact that restricted social contact had on gastrointestinal diseases (**Figure 3**, Table S5 in the **Online Supplementary Document**) stands in striking contrast to the significant reduction of enteric infections observed in industrialized countries during the pandemic [47-49]. This indicates that some local factors, for instance reduced access to clean water, might have counteracted any effect of social distancing on the transmission of enteric pathogens in Kenya. In one survey, 39% of respondents indicated that COVID-19 made it more difficult getting drinking water, especially among people who lost their jobs and who faced financial difficulties [80]. The water service modality also changed, with rural residents reporting a 20% reduction in piped water usage, and a 19% increase in river / pond water usage [80]. Hence, while in industrialized countries with full access to clean water, the restricted social contacts resulted in a reduction of enteric infections, in LMICs as Kenya, it is possible that the reduced access to clean water, and increased use of unsafe water sources might have increased the prevalence of waterborne intestinal infections, masking any possible effect of the COVID-19 measures on person-to-person transmission of enteric pathogens (**Figure 3**, Table S5 in the **Online Supplementary Document**).

Despite predictions of increased malaria morbidity and mortality in Africa as an indirect effect of the COVID-19 pandemic on malaria control interventions [81,82], we found that, according to both the official facility-based records and our Diaries, malaria incidence declined significantly after the onset of the pandemic in Kisumu and Kakamega until August 2020 (**Figure 4**, Table S6 in the **Online Supplementary Document**). Between September and November 2020, self-reported malaria / fever incidence remains low, especially for children, while the official data from Kisumu and Kakamega showed a peak of malaria cases that was higher than in the years 2018 and 2019 for the same period (**Figure 4**). Interestingly, other malaria-endemic African countries (Democratic Republic of Congo, Ghana, Rwanda, Sierra Leone, Uganda) have also reported reduced numbers of malaria cases during the lockdown months [83-86], while low endemic countries reported higher numbers of malaria cases and deaths during the pandemic (Zimbabwe, Zambia) [83].

Several factors may have played a role in the observed decline of malaria cases observed in facilities in highly endemic countries, e.g. reduced health care utilization due to travel restrictions or fear of COVID-19. However, the self-reported diaries data indicate that indeed less malaria / fever cases occurred in these households, suggesting that the lower malaria incidence during mobility restrictions in other countries might also reflect real declines. For instance, children could have played less frequently outside during malaria evening transmission moments due to curfews. Also, since malaria transmission frequently results from migration flows of infected individuals across different regions [83,87], the stringent mobility restrictions during the first months of the pandemic may have affected the migration of malaria parasites between distant communities. Supporting this hypothesis is the fact that both the DHIS2 and the diaries data observed a reduction also during the first malaria / rainy season March-May 2020, while rainfall was actually above normal in 2020 during these months [88]. This period coincided with the most stringent months of COVID-19 measures in Kenya, with limited migration between distant communities. This would also explain why the DHIS2 data showed a peak of malaria cases during the second rainy season September-December 2020, when many adults started to travel again in search for an income. We do not observe this September-November peak in our Diaries’ households in 2020, potentially due to their different demographic characteristics compared to the whole Kisumu / Kakamega population. Our households were formed by young families who may have used bed nets to protect their children as advised by community health care workers. Also, in September 2019, Kakamega and Kisumu participated in a pilot study of the RTS,S malaria vaccine that was provided to children younger than two years old [89]. Our data from Kakamega show that 51% of the children below six in our households had received the vaccine and hence were protected against severe / symptomatic malaria (we do not have data for Kisumu). All in all, these findings, contradict the predictions made by international health organizations about the impact of the pandemic on mosquito-borne diseases in Africa and warrant further investigation.

We also captured in our diaries (not shown by DHIS2 data) a higher prevalence of NCDs in both the paediatric and adult population during the pandemic. On the one hand, this could be a result of discontinued care during the first months of the pandemic, and consequent exacerbation of these conditions and “catch-up care” later on (e.g. dental health, or conditions that required surgery). On the other hand, the most frequently reported NCDs during these months were headache, backaches, and general body weakness. Pain, regardless of the anatomic side, is associated with depression and anxiety [90]. Since these conditions were highly prevalent during the pandemic among low-income populations across Sub-Saharan Africa [91], the NCD symptoms may have reflected increased mental health problems or mild COVID-19.

Interestingly, we did not observe a significant decline in health-seeking behaviour at formal facilities during the pandemic in our households. However, we observed a temporary shift from health care facilities to (qualified) pharmacists / medicine vendors. Since the latter are mostly of private ownership, this shift may have been triggered by fear of acquiring COVID-19 at larger health care centres, rather than by financial constraints. On the other hand, reduced transport possibilities to more far-away facilities due to curfew could have played a role. Outside the COVID-19 waves, patients returned to their usual health-seeking behaviour. Part of the Diaries households in Kakamega received access to subsidized health insurance from July 2020 onwards; this may have helped some households to seek formal health care during the pandemic when needed.

### Strengths and limitations

This study has several limitations. Although the information of our Diaries mostly aligns with facility-based data, it is important to consider the demographic characteristics of our young households before extrapolating our findings to the Kisumu / Kakamega population. Also, although the diaries interviewers had a good rapport with the respondents and there is no reason to believe they would withhold/make up any symptom/medical condition, it is always possible that such verbal reports lack accuracy, also taking into consideration that no diagnostic tests/medical examination could confirm the reported conditions. On the other hand, the possibility of recalling bias is very low since the interviews were performed weekly, and for instance in the case of NCDs, our diaries captured more information than the DHIS2 data.

### Implication for policy and future research

We provide evidence that the lockdown measures decreased the incidence of respiratory pathogens, as well as gastrointestinal and malaria infections in this rural, low-income setting in Western Kenya. This shows that non-pharmaceutical containment measures were also effective in a limited-resource setting in reducing the spread of infections. This can guide interventions to control future epidemics of airborne pathogens in this Kenyan region, but also in similar settings in other LMICs. However, the local context matters: the population should have access to the necessary resources and infrastructure to abide with the lockdown measures. In particular, improved access to water and sanitation is urgently needed since the lockdown interventions only modestly impacted the incidence of intestinal illnesses. Research is needed to better understand the impact of mobility on malaria transmission, and to assess the impact of the introduction of malaria vaccines on the incidence of symptomatic / severe malaria in the paediatric population. Furthermore, we highlight the vulnerability of low-income families in times of pandemics: the increased incidence of respiratory infections among the adults (during the COVID-19 waves) indicate the challenges for informal workers/low-income families to follow such stringent mobility measures, while the NCD symptoms are suggestive of heightened depression / anxiety during the pandemic or mild COVID-19.

## CONCLUSIONS

Health diaries are a reliable source of information that allow capturing the dynamics of both infectious and NCDs, and the health-seeking behaviour of vulnerable populations in times of public health emergencies. They allow for a better interpretation of the observed facility-based data, and are hence of great value for evidence-based decisions and policy-making. Granular Diaries data, triangulated with DHIS2 health facilities data indicate that COVID-19 (measures) coincided with reduced incidence of respiratory and enteric diseases, and malaria / fever in particular for children; while report of NCDs increased according to Diaries data. No significant reduction in health care utilization conditional on seeking care was observed, apart from temporary shifts to private retail pharmacies.

## Additional material


Online Supplementary Document
