# Supplementary material for: Reduced incidence of respiratory, gastrointestinal and malaria infections among children during the COVID-19 pandemic in Western Kenya: An analysis of facility-based and weekly diaries data
Source: J Glob Health. 2023 Jul 14;13:06024. doi: 10.7189/jogh.13.06024 (PMC10346133; doi:10.7189/jogh.13.06024)
Supplement: Online Supplementary Document [file jogh-13-06024-s001.pdf]

Supplementary Materials

Reduced Incidence of Infectious Diseases Among Children During the COVID-19 Pandemic in Western Kenya: An Analysis of Facility-Based and Weekly Diaries Data

Gloria P. Gómez-Pérez, Richard de Groot, Amanuel Abajobir, Carol Wainaina, Tobias F. Rinke de Wit, Estelle Sidze, Menno Pradhan, Wendy Janssens

This file contains the eight **Supplementary Tables**, **Supplementary Methods** and **Supplementary Figures** refer to in the manuscript.

Supplementary Tables

Supplementary Table 1: DHIS2 90 kinds of illnesses or health problems, and assigned category.

	Description of diagnosis	Category
1	Abortion	SRHR
2	All other diseases	Other
3	Amoebiasis	Gastrointestinal
4	Anaemia cases	NCD
5	Arthritis, Joint pains etc.	NCD
6	Asthma	NCD
7	Autism	*(Not applicable – not a disease)*
8	Brucellosis	Other CD
9	Burns	Accident
10	Cardiovascular conditions	NCD
11	Central Nervous System Conditions	NCD
12	Cerebral Palsy	NCD
13	Chicken Pox	Respiratory/airborne
14	Chikungunya	Other CD
15	Cholera	Gastrointestinal
16	Confirmed Malaria (only Positive cases)	Malaria
17	Cutaneous leishmaniasis	Other CD
18	Deaths due to Road Traffic Injuries	Accident
19	Dengue fever	Other CD
20	Dental Disorders	NCD
21	Diabetes	NCD
22	Diarrhoea	Gastrointestinal

23	Diarrhoea with no dehydration	Gastrointestinal
24	Diarrhoea with severe dehydration	Gastrointestinal
25	Diarrhoea with some dehydration	Gastrointestinal
26	Disease of Puerperium and Childbirth	SRHR
27	Disease of the skin	NCD
28	Dog Bites	Accident
29	Down's syndrome	*(Not applicable – not a disease)*
30	Down's syndromes	*(Not applicable – not a disease)*
31	Dysentery (Bloody Diarrhoea)	Gastrointestinal
32	Ear Infections/ Conditions	Other CD
33	Epilepsy	NCD
34	Eye Infections	Other CD
35	Fistula (Birth related)	SRHR
36	Gastroenteritis	Gastrointestinal
37	Hepatitis	Other CD
38	Hypertension	NCD
39	Hypoxaemia (Spo2<90%)	NCD
40	Intestinal worms	Gastrointestinal
41	Jiggers Infestation	Other CD
42	Leishmaniaisis (Kalazar)	Other CD
43	Lower Respiratory Tract Infections	Respiratory/airborne
44	Malaria in pregnancy	Malaria
45	Malnutrition	NCD
46	Measles	Respiratory/airborne
47	Meningococcal Meningitis	Respiratory/airborne
48	Mumps	Respiratory/airborne
49	Muscular skeletal conditions	NCD
50	Neonatal Sepsis	SRHR
51	Neonatal Tetanus	SRHR
52	Neoplasms	NCD
53	Other Dis. Of Respiratory System	NCD
54	Other Bites	Accident
55	Other Central Nervous System Conditions	NCD
56	Other Convulsive Disorders	NCD
57	Other Eye conditions	NCD
58	Other injuries	Accident
59	Other Meningitis	Respiratory/airborne
60	Overweight (BMI >25)	NCD
61	Physical Disability	NCD
62	Plague	Other CD
63	Pneumonia	Respiratory/airborne
64	Pneumonia	Respiratory/airborne
65	Poisoning	Accident
66	Poliomyelitis (AFP)	Other CD
67	Presumed Tuberculosis	Respiratory/airborne

68	Referrals from Community Unit	*(Not applicable – not a disease)*
69	Referrals to Community Unit	*(Not applicable – not a disease)*
70	Rheumatic Fever	NCD
71	Rickets	NCD
72	Rift valley fever	Other CD
73	Road Traffic Injuries	Accident
74	Severe pneumonia	Respiratory/airborne
75	Sexual Violence	Accident
76	Sexually Transmitted Infections	Other CD
77	Snake Bites	Accident
78	Suspected Malaria	Malaria
79	Suspected anthrax	Other CD
80	Suspected Childhood Cancers	NCD
81	Tested for Malaria	Malaria
82	Tetanus	Other CD
83	Tonsilitis	Respiratory/airborne
84	Tryponosomiasis	Other CD
85	Typhoid fever	Other CD
86	Upper Respiratory Tract Infections	Respiratory/airborne
87	Urinary Tract Infection	NCD
88	Violence related injuries	Accident
89	Viral Haemorrhagic Fever	Other CD
90	Yellow fever cases	Other CD

Abbreviations: CD, communicable diseases; NCD, non-communicable diseases; SRHR, Sexual and Reproductive Health and Rights.

Supplementary Table 2: 47 health problems/symptoms recorded in the Diaries, and assigned category.

	Description of symptom/problem	Category
1	Fever/malaria	Malaria
2	Typhoid fever	Other CD
3	Flu/cold/cough	Respiratory/airborne
4	Chest pain (non-respiratory)	NCD
5	Respiratory problems	Respiratory/airborne
6	Cancer	NCD
7	Diarrhoea/stomach problems/vomit	Gastrointestinal
8	Traffic accident	Accident
9	Cuts/bleeding/wounds (injury)	Accident
10	Burns	Accident
11	Skin problems/allergy/itching	NCD
12	Genital discomfort	SRHR
13	Urinary infection	NCD
14	Hypertension	NCD
15	Diabetes	NCD
16	Headache	NCD
17	Backache/neck	NCD
18	Arthritis/rheumatism/joint pains	NCD
19	Hernia	NCD
20	Broken bones/sprain	Accident
21	Poor vision	NCD
22	Dental/mouth problems	NCD
23	Anaemia	NCD
24	Worms	Gastrointestinal
25	Shortness of breath	Respiratory/airborne
26	Malaria	Malaria
27	Measles	Respiratory/airborne
28	Eye problems	NCD
29	Ear problems	NCD
30	General body weakness	NCD
31	Epilepsy	NCD
32	Covid-19	Respiratory/airborne
33	Family planning/ANC/post-delivery	SRHR
34	Pregnancy-related problems	SRHR
35	Abdominal pain	Gastrointestinal
36	Bite/stung by animal	Accident
37	Delivery/birth	SRHR
38	Faint/lost consciousness	NCD
39	Goitre	NCD
40	Heart problems	NCD
41	Hydrocephalus	NCD
42	Jaundice	NCD

43	Leg problems	NCD
44	Nervous system	NCD
45	Tuberculosis	Respiratory/airborne
46	Other NCD	NCD
47	Other CD	Other CD

Abbreviations: CD, communicable diseases; NCD, non-communicable diseases; SRHR, Sexual and Reproductive Health and Rights.

Supplementary Table 3: Change in share of any health problems after 17 March, compared to the period 4 February - 16 March.

	All members	Children	Adults
24 March 2020 - 30 March 2020	-0.019*	-0.015	-0.028*
	(0.011)	(0.014)	(0.015)
31 March 2020 - 06 April 2020	-0.039***	-0.044***	-0.029*
	(0.009)	(0.012)	(0.015)
07 April 2020 - 13 April 2020	-0.059***	-0.070***	-0.039***
	(0.008)	(0.010)	(0.015)
14 April 2020 - 20 April 2020	-0.039***	-0.042***	-0.032**
	(0.009)	(0.012)	(0.014)
21 April 2020 - 27 April 2020	-0.051***	-0.063***	-0.027*
	(0.010)	(0.013)	(0.016)
28 April 2020 - 04 May 2020	-0.044***	-0.059***	-0.015
	(0.009)	(0.011)	(0.016)
05 May 2020 - 11 May 2020	0.020*	-0.010	0.078***
	(0.011)	(0.012)	(0.019)
12 May 2020 - 18 May 2020	-0.027***	-0.067***	0.034**
	(0.010)	(0.011)	(0.017)
19 May 2020 - 25 May 2020	-0.030***	-0.060***	0.019
	(0.009)	(0.011)	(0.016)
26 May 2020 - 01 June 2020	-0.038***	-0.058***	-0.005
	(0.009)	(0.011)	(0.014)
16 June 2020 - 22 June 2020	-0.001	-0.034***	0.051***
	(0.011)	(0.013)	(0.017)
23 June 2020 - 29 June 2020	0.005	-0.034**	0.068***
	(0.012)	(0.013)	(0.019)
30 June 2020 - 06 July 2020	-0.002	-0.030**	0.045***
	(0.011)	(0.013)	(0.017)
07 July 2020 - 13 July 2020	0.002	-0.020	0.040**
	(0.012)	(0.015)	(0.017)
14 July 2020 - 20 July 2020	0.018*	-0.003	0.053***
	(0.011)	(0.014)	(0.017)
21 July 2020 - 27 July 2020	-0.012	-0.031**	0.019
	(0.011)	(0.013)	(0.016)
28 July 2020 - 03 August 2020	-0.029**	-0.048***	0.005
	(0.011)	(0.013)	(0.015)
04 August 2020 - 10 August 2020	-0.027***	-0.073***	0.040**
	(0.010)	(0.011)	(0.018)
11 August 2020 - 17 August 2020	-0.007	-0.019*	0.014
	(0.010)	(0.012)	(0.017)
18 August 2020 - 24 August 2020	-0.039***	-0.086***	0.030*
	(0.010)	(0.010)	(0.016)
25 August 2020 - 31 August 2020	-0.044***	-0.063***	-0.012
	(0.009)	(0.011)	(0.014)
01 September 2020 - 07 September 2020	-0.051***	-0.095***	0.021
	(0.009)	(0.009)	(0.018)
08 September 2020 - 14 September 2020	-0.051***	-0.057***	-0.038***
	(0.008)	(0.011)	(0.014)
15 September 2020 - 21 September 2020	-0.047***	-0.062***	-0.020
	(0.009)	(0.011)	(0.014)

22 September 2020 - 28 September 2020	-0.039***	-0.056***	-0.009
	(0.009)	(0.010)	(0.015)
29 September 2020 - 05 October 2020	-0.050***	-0.067***	-0.018
	(0.009)	(0.011)	(0.014)
06 October 2020 - 12 October 2020	-0.029***	-0.048***	0.005
	(0.009)	(0.011)	(0.015)
13 October 2020 - 19 October 2020	-0.030***	-0.035***	-0.018
	(0.009)	(0.012)	(0.015)
20 October 2020 - 26 October 2020	-0.047***	-0.059***	-0.025*
	(0.009)	(0.011)	(0.014)
27 October 2020 - 02 November 2020	-0.050***	-0.104***	0.031*
	(0.009)	(0.009)	(0.017)
03 November 2020 - 09 November 2020	-0.030***	-0.050***	0.007
	(0.010)	(0.012)	(0.016)
10 November 2020 - 16 November 2020	-0.010	-0.021	0.011
	(0.012)	(0.013)	(0.016)
17 November 2020 - 23 November 2020	-0.065***	-0.106***	0.001
	(0.008)	(0.009)	(0.016)
24 November 2020 - 30 November 2020	-0.031***	-0.044***	-0.005
	(0.011)	(0.013)	(0.016)
Mean between 4 February - 16 March	0.122	0.127	0.114
Joint significance of all post-COVID weeks (p-value)	0.000	0.000	0.000
Observations	55,923	34,719	21,204
R^2	0.006	0.011	0.008
Household FE	YES	YES	YES

Table presents estimates from household fixed-effects regressions, with standard errors clustered at the household level.
* $P < 0.1$; ** $P < 0.05$; *** $P < 0.01$.

Supplementary Table 4: Change in share of Respiratory/air-borne diseases after 17 March, compared to the period 4 February - 16 March

	All members	Children	Adults
24 March 2020 - 30 March 2020	0.006	0.007	0.002
	(0.008)	(0.011)	(0.009)
31 March 2020 - 06 April 2020	-0.009	-0.020**	0.010
	(0.007)	(0.009)	(0.010)
07 April 2020 - 13 April 2020	-0.029***	-0.037***	-0.013**
	(0.005)	(0.007)	(0.007)
14 April 2020 - 20 April 2020	-0.018***	-0.029***	0.003
	(0.007)	(0.008)	(0.008)
21 April 2020 - 27 April 2020	-0.025***	-0.035***	-0.006
	(0.006)	(0.009)	(0.008)
28 April 2020 - 04 May 2020	-0.028***	-0.044***	0.001
	(0.006)	(0.007)	(0.008)
05 May 2020 - 11 May 2020	0.004	-0.007	0.024**
	(0.008)	(0.010)	(0.011)
12 May 2020 - 18 May 2020	-0.024***	-0.042***	0.007
	(0.006)	(0.007)	(0.009)
19 May 2020 - 25 May 2020	-0.026***	-0.042***	0.002
	(0.005)	(0.006)	(0.007)
26 May 2020 - 01 June 2020	-0.032***	-0.042***	-0.013*
	(0.005)	(0.007)	(0.007)
16 June 2020 - 22 June 2020	-0.016**	-0.027***	0.006
	(0.007)	(0.008)	(0.009)
23 June 2020 - 29 June 2020	-0.005	-0.024**	0.028**
	(0.008)	(0.010)	(0.012)
30 June 2020 - 06 July 2020	-0.006	-0.018**	0.017
	(0.007)	(0.009)	(0.010)
07 July 2020 - 13 July 2020	0.006	0.001	0.019*
	(0.010)	(0.013)	(0.010)
14 July 2020 - 20 July 2020	0.010	0.004	0.023**
	(0.008)	(0.011)	(0.010)
21 July 2020 - 27 July 2020	-0.013*	-0.030***	0.016
	(0.007)	(0.009)	(0.010)
28 July 2020 - 03 August 2020	0.001	-0.015	0.031***
	(0.009)	(0.011)	(0.010)
04 August 2020 - 10 August 2020	-0.015**	-0.034***	0.019*
	(0.007)	(0.008)	(0.010)
11 August 2020 - 17 August 2020	0.005	-0.002	0.017*
	(0.008)	(0.010)	(0.010)
18 August 2020 - 24 August 2020	-0.021***	-0.043***	0.017*
	(0.006)	(0.007)	(0.010)
25 August 2020 - 31 August 2020	-0.019***	-0.032***	0.003
	(0.006)	(0.008)	(0.008)
01 September 2020 - 07 September 2020	-0.035***	-0.054***	0.001
	(0.006)	(0.006)	(0.011)
08 September 2020 - 14 September 2020	-0.020***	-0.028***	-0.006
	(0.006)	(0.008)	(0.006)
15 September 2020 - 21 September 2020	-0.015**	-0.023***	0.001
	(0.007)	(0.009)	(0.008)

22 September 2020 - 28 September 2020	-0.017***	-0.030***	0.007
	(0.006)	(0.007)	(0.009)
29 September 2020 - 05 October 2020	-0.013*	-0.025***	0.010
	(0.008)	(0.009)	(0.009)
06 October 2020 - 12 October 2020	-0.008	-0.017**	0.008
	(0.007)	(0.009)	(0.009)
13 October 2020 - 19 October 2020	-0.008	-0.011	-0.001
	(0.007)	(0.009)	(0.008)
20 October 2020 - 26 October 2020	-0.016**	-0.023***	-0.002
	(0.007)	(0.009)	(0.008)
27 October 2020 - 02 November 2020	-0.021***	-0.052***	0.029**
	(0.007)	(0.006)	(0.012)
03 November 2020 - 09 November 2020	0.001	-0.012	0.025**
	(0.008)	(0.009)	(0.011)
10 November 2020 - 16 November 2020	0.017	0.013	0.026**
	(0.010)	(0.012)	(0.011)
17 November 2020 - 23 November 2020	-0.026***	-0.051***	0.016
	(0.006)	(0.006)	(0.010)
24 November 2020 - 30 November 2020	-0.001	-0.006	0.009
	(0.007)	(0.010)	(0.009)
Mean between 4 February - 16 March	0.046	0.057	0.025
Joint significance of all post-COVID weeks (p-value)	0.000	0.000	0.000
Observations	55,923	34,719	21,204
R^2	0.005	0.009	0.004
Household FE	YES	YES	YES

Table presents estimates from household fixed-effects regressions, with standard errors clustered at the household level.
* $P < 0.1$; ** $P < 0.05$; *** $P < 0.01$.

Supplementary Table 5: Change in share of Gastrointestinal diseases after 17 March, compared to the period 4 February - 16 March

	All members	Children	Adults
24 March 2020 - 30 March 2020	-0.004	-0.004	-0.004
	(0.003)	(0.004)	(0.004)
31 March 2020 - 06 April 2020	-0.006*	-0.004	-0.009***
	(0.003)	(0.005)	(0.002)
07 April 2020 - 13 April 2020	-0.006**	-0.009***	-0.000
	(0.003)	(0.003)	(0.005)
14 April 2020 - 20 April 2020	-0.005**	-0.007**	-0.002
	(0.003)	(0.003)	(0.004)
21 April 2020 - 27 April 2020	-0.006**	-0.008***	-0.002
	(0.003)	(0.003)	(0.005)
28 April 2020 - 04 May 2020	-0.005*	-0.004	-0.004
	(0.002)	(0.004)	(0.003)
05 May 2020 - 11 May 2020	0.001	-0.000	0.004
	(0.003)	(0.004)	(0.006)
12 May 2020 - 18 May 2020	0.004	0.002	0.008
	(0.004)	(0.004)	(0.006)
19 May 2020 - 25 May 2020	-0.003	-0.005	0.000
	(0.003)	(0.004)	(0.004)
26 May 2020 - 01 June 2020	0.002	-0.006	0.014**
	(0.004)	(0.004)	(0.007)
16 June 2020 - 22 June 2020	0.003	-0.001	0.010*
	(0.003)	(0.004)	(0.006)
23 June 2020 - 29 June 2020	0.005	0.000	0.013**
	(0.004)	(0.005)	(0.006)
30 June 2020 - 06 July 2020	0.004	0.004	0.003
	(0.003)	(0.004)	(0.005)
07 July 2020 - 13 July 2020	0.000	-0.000	0.002
	(0.003)	(0.004)	(0.004)
14 July 2020 - 20 July 2020	0.000	-0.001	0.003
	(0.003)	(0.004)	(0.005)
21 July 2020 - 27 July 2020	0.001	-0.001	0.004
	(0.004)	(0.004)	(0.005)
28 July 2020 - 03 August 2020	-0.004	-0.007*	0.002
	(0.003)	(0.003)	(0.005)
04 August 2020 - 10 August 2020	0.003	-0.003	0.011
	(0.004)	(0.003)	(0.007)
11 August 2020 - 17 August 2020	-0.000	0.001	-0.002
	(0.003)	(0.004)	(0.004)
18 August 2020 - 24 August 2020	0.000	-0.006**	0.010*
	(0.003)	(0.003)	(0.006)
25 August 2020 - 31 August 2020	-0.004*	-0.007**	0.000
	(0.002)	(0.003)	(0.004)
01 September 2020 - 07 September 2020	0.003	-0.005	0.016*
	(0.005)	(0.003)	(0.009)
08 September 2020 - 14 September 2020	-0.004	-0.003	-0.005
	(0.003)	(0.004)	(0.003)
15 September 2020 - 21 September 2020	-0.003	-0.004	0.000
	(0.003)	(0.003)	(0.005)

22 September 2020 - 28 September 2020	-0.001	-0.001	0.001
	(0.003)	(0.005)	(0.004)
29 September 2020 - 05 October 2020	-0.005	-0.007	0.000
	(0.004)	(0.005)	(0.004)
06 October 2020 - 12 October 2020	0.001	0.000	0.002
	(0.003)	(0.004)	(0.005)
13 October 2020 - 19 October 2020	0.003	0.003	0.002
	(0.003)	(0.005)	(0.005)
20 October 2020 - 26 October 2020	-0.002	-0.003	0.001
	(0.003)	(0.004)	(0.005)
27 October 2020 - 02 November 2020	-0.002	-0.003	-0.001
	(0.003)	(0.004)	(0.004)
03 November 2020 - 09 November 2020	-0.007***	-0.009***	-0.003
	(0.002)	(0.003)	(0.004)
10 November 2020 - 16 November 2020	-0.002	-0.008***	0.008
	(0.003)	(0.003)	(0.006)
17 November 2020 - 23 November 2020	-0.004	-0.007**	0.003
	(0.003)	(0.003)	(0.005)
24 November 2020 - 30 November 2020	-0.003	-0.008***	0.005
	(0.003)	(0.003)	(0.005)
Mean between 4 February - 16 March	0.011	0.012	0.009
Joint significance of all post-COVID weeks (p-value)	0.003	0.002	0.000
Observations	55,923	34,719	21,204
R^2	0.001	0.001	0.003
Household FE	YES	YES	YES

Table presents estimates from household fixed-effects regressions, with standard errors clustered at the household level.
* $P < 0.1$; ** $P < 0.05$; *** $P < 0.01$.

Supplementary Table 6: Change in share of Malaria after 17 March, compared to the period 4 February - 16 March

	All members	Children	Adults
24 March 2020 - 30 March 2020	-0.013*	-0.012	-0.018**
	(0.007)	(0.010)	(0.008)
31 March 2020 - 06 April 2020	-0.022***	-0.028***	-0.013*
	(0.007)	(0.009)	(0.008)
07 April 2020 - 13 April 2020	-0.030***	-0.041***	-0.010
	(0.006)	(0.008)	(0.009)
14 April 2020 - 20 April 2020	-0.012*	-0.018**	-0.001
	(0.007)	(0.009)	(0.009)
21 April 2020 - 27 April 2020	-0.022***	-0.037***	0.005
	(0.007)	(0.009)	(0.010)
28 April 2020 - 04 May 2020	-0.022***	-0.035***	0.002
	(0.007)	(0.009)	(0.009)
05 May 2020 - 11 May 2020	-0.011	-0.017*	0.002
	(0.007)	(0.009)	(0.010)
12 May 2020 - 18 May 2020	-0.030***	-0.061***	0.021**
	(0.006)	(0.007)	(0.010)
19 May 2020 - 25 May 2020	-0.021***	-0.039***	0.011
	(0.006)	(0.008)	(0.009)
26 May 2020 - 01 June 2020	-0.034***	-0.045***	-0.014*
	(0.007)	(0.009)	(0.007)
16 June 2020 - 22 June 2020	-0.008	-0.026***	0.024**
	(0.007)	(0.009)	(0.011)
23 June 2020 - 29 June 2020	-0.016**	-0.036***	0.019*
	(0.007)	(0.009)	(0.010)
30 June 2020 - 06 July 2020	-0.020***	-0.028***	-0.004
	(0.006)	(0.009)	(0.008)
07 July 2020 - 13 July 2020	-0.023***	-0.038***	0.003
	(0.006)	(0.008)	(0.009)
14 July 2020 - 20 July 2020	-0.015**	-0.026***	0.006
	(0.007)	(0.009)	(0.009)
21 July 2020 - 27 July 2020	-0.031***	-0.037***	-0.016**
	(0.006)	(0.009)	(0.007)
28 July 2020 - 03 August 2020	-0.032***	-0.042***	-0.013*
	(0.006)	(0.009)	(0.007)
04 August 2020 - 10 August 2020	-0.036***	-0.066***	0.013
	(0.005)	(0.006)	(0.010)
11 August 2020 - 17 August 2020	-0.026***	-0.037***	-0.006
	(0.006)	(0.008)	(0.008)
18 August 2020 - 24 August 2020	-0.042***	-0.066***	-0.004
	(0.006)	(0.007)	(0.009)
25 August 2020 - 31 August 2020	-0.037***	-0.053***	-0.010
	(0.005)	(0.007)	(0.007)
01 September 2020 - 07 September 2020	-0.042***	-0.065***	0.001
	(0.005)	(0.006)	(0.008)
08 September 2020 - 14 September 2020	-0.034***	-0.042***	-0.019***
	(0.005)	(0.008)	(0.006)
15 September 2020 - 21 September 2020	-0.041***	-0.048***	-0.028***
	(0.006)	(0.008)	(0.005)

22 September 2020 - 28 September 2020	-0.033***	-0.044***	-0.015**
	(0.005)	(0.007)	(0.007)
29 September 2020 - 05 October 2020	-0.045***	-0.061***	-0.017**
	(0.005)	(0.007)	(0.007)
06 October 2020 - 12 October 2020	-0.034***	-0.043***	-0.018***
	(0.006)	(0.007)	(0.007)
13 October 2020 - 19 October 2020	-0.033***	-0.042***	-0.017***
	(0.006)	(0.008)	(0.006)
20 October 2020 - 26 October 2020	-0.033***	-0.047***	-0.010
	(0.006)	(0.008)	(0.008)
27 October 2020 - 02 November 2020	-0.039***	-0.071***	0.012
	(0.005)	(0.006)	(0.010)
03 November 2020 - 09 November 2020	-0.028***	-0.041***	-0.006
	(0.007)	(0.008)	(0.008)
10 November 2020 - 16 November 2020	-0.031***	-0.037***	-0.019***
	(0.006)	(0.007)	(0.007)
17 November 2020 - 23 November 2020	-0.046***	-0.070***	-0.004
	(0.005)	(0.006)	(0.008)
24 November 2020 - 30 November 2020	-0.035***	-0.043***	-0.019***
	(0.006)	(0.008)	(0.007)
Mean between 4 February - 16 March	0.060	0.075	0.033
Joint significance of all post-COVID weeks (p-value)	0.000	0.000	0.000
Observations	55,923	34,719	21,204
R^2	0.005	0.010	0.005
Household FE	YES	YES	YES

Table presents estimates from household fixed-effects regressions, with standard errors clustered at the household level.
* $P < 0.1$; ** $P < 0.05$; *** $P < 0.01$.

Supplementary Table 7: Change in share of NCDs after 17 March, compared to the period 4 February - 16 March

	All members	Children	Adults
24 March 2020 - 30 March 2020	-0.008*	-0.003	-0.015*
	(0.004)	(0.005)	(0.009)
31 March 2020 - 06 April 2020	0.003	0.006	-0.001
	(0.006)	(0.007)	(0.009)
07 April 2020 - 13 April 2020	-0.002	-0.001	-0.003
	(0.005)	(0.005)	(0.009)
14 April 2020 - 20 April 2020	0.004	0.007	-0.001
	(0.005)	(0.006)	(0.009)
21 April 2020 - 27 April 2020	0.003	0.006	0.000
	(0.005)	(0.006)	(0.011)
28 April 2020 - 04 May 2020	0.011*	0.007	0.017
	(0.005)	(0.006)	(0.011)
05 May 2020 - 11 May 2020	0.026***	0.009	0.059***
	(0.007)	(0.006)	(0.014)
12 May 2020 - 18 May 2020	0.019***	0.002	0.040***
	(0.006)	(0.006)	(0.011)
19 May 2020 - 25 May 2020	0.018***	0.009	0.029**
	(0.006)	(0.007)	(0.012)
26 May 2020 - 01 June 2020	0.017***	0.012*	0.023**
	(0.006)	(0.007)	(0.011)
16 June 2020 - 22 June 2020	0.034***	0.014**	0.060***
	(0.007)	(0.007)	(0.012)
23 June 2020 - 29 June 2020	0.034***	0.019**	0.054***
	(0.008)	(0.008)	(0.014)
30 June 2020 - 06 July 2020	0.030***	0.012**	0.057***
	(0.006)	(0.006)	(0.013)
07 July 2020 - 13 July 2020	0.024***	0.013*	0.040***
	(0.006)	(0.007)	(0.012)
14 July 2020 - 20 July 2020	0.044***	0.029***	0.065***
	(0.008)	(0.009)	(0.014)
21 July 2020 - 27 July 2020	0.026***	0.024***	0.025**
	(0.006)	(0.007)	(0.010)
28 July 2020 - 03 August 2020	0.006	0.003	0.012
	(0.005)	(0.005)	(0.010)
04 August 2020 - 10 August 2020	0.015**	0.005	0.025**
	(0.006)	(0.006)	(0.010)
11 August 2020 - 17 August 2020	0.018***	0.012*	0.029***
	(0.006)	(0.006)	(0.011)
18 August 2020 - 24 August 2020	0.016***	-0.001	0.035***
	(0.005)	(0.005)	(0.010)
25 August 2020 - 31 August 2020	0.016***	0.012*	0.021**
	(0.006)	(0.007)	(0.010)
01 September 2020 - 07 September 2020	0.013**	0.004	0.025**
	(0.006)	(0.006)	(0.010)
08 September 2020 - 14 September 2020	0.009*	0.009	0.010
	(0.005)	(0.006)	(0.010)
15 September 2020 - 21 September 2020	0.006	-0.001	0.018*
	(0.006)	(0.006)	(0.011)

22 September 2020 - 28 September 2020	0.008	0.002	0.019*
	(0.006)	(0.005)	(0.011)
29 September 2020 - 05 October 2020	0.006	-0.001	0.018*
	(0.005)	(0.005)	(0.011)
06 October 2020 - 12 October 2020	0.016***	0.003	0.040***
	(0.005)	(0.005)	(0.012)
13 October 2020 - 19 October 2020	0.012**	0.006	0.022**
	(0.006)	(0.005)	(0.011)
20 October 2020 - 26 October 2020	0.003	0.001	0.008
	(0.005)	(0.005)	(0.010)
27 October 2020 - 02 November 2020	0.007	-0.006	0.023**
	(0.005)	(0.005)	(0.011)
03 November 2020 - 09 November 2020	0.015**	0.008	0.026**
	(0.006)	(0.006)	(0.011)
10 November 2020 - 16 November 2020	0.016***	0.015**	0.018
	(0.006)	(0.007)	(0.011)
17 November 2020 - 23 November 2020	0.007	-0.008**	0.026**
	(0.005)	(0.004)	(0.011)
24 November 2020 - 30 November 2020	0.017**	0.010	0.030**
	(0.007)	(0.007)	(0.013)
Mean between 4 February - 16 March	0.024	0.016	0.038
Joint significance of all post-COVID weeks (p-value)	0.000	0.000	0.000
Observations	55,923	34,719	21,204
R^2	0.004	0.003	0.007
Household FE	YES	YES	YES

Table presents estimates from household fixed-effects regressions, with standard errors clustered at the household level.
* $P < 0.1$; ** $P < 0.05$; *** $P < 0.01$.

Supplementary Table 8: Change in share of seeking formal care for a health problem after 17 March, compared to the period 4 February - 16 March

	All members	Children	Adults
24 March 2020 - 30 March 2020	0.012	0.004	-0.003
	(0.067)	(0.074)	(0.144)
31 March 2020 - 06 April 2020	-0.080	-0.109	-0.167
	(0.065)	(0.093)	(0.108)
07 April 2020 - 13 April 2020	0.089	0.044	0.097
	(0.073)	(0.089)	(0.146)
14 April 2020 - 20 April 2020	0.005	0.013	-0.069
	(0.067)	(0.090)	(0.112)
21 April 2020 - 27 April 2020	-0.066	-0.107	-0.062
	(0.074)	(0.097)	(0.127)
28 April 2020 - 04 May 2020	-0.169**	-0.192*	-0.139
	(0.071)	(0.101)	(0.119)
05 May 2020 - 11 May 2020	-0.015	0.001	0.042
	(0.081)	(0.104)	(0.106)
12 May 2020 - 18 May 2020	-0.051	0.070	-0.013
	(0.066)	(0.092)	(0.097)
19 May 2020 - 25 May 2020	-0.060	-0.049	0.002
	(0.068)	(0.124)	(0.090)
26 May 2020 - 01 June 2020	-0.076	-0.029	-0.050
	(0.095)	(0.122)	(0.136)
16 June 2020 - 22 June 2020	-0.049	-0.109	0.129
	(0.069)	(0.101)	(0.086)
23 June 2020 - 29 June 2020	-0.156***	-0.082	-0.133
	(0.054)	(0.081)	(0.082)
30 June 2020 - 06 July 2020	-0.089	-0.065	-0.112
	(0.056)	(0.076)	(0.077)
07 July 2020 - 13 July 2020	-0.028	0.002	-0.095
	(0.057)	(0.072)	(0.081)
14 July 2020 - 20 July 2020	-0.157***	-0.174*	-0.086
	(0.060)	(0.091)	(0.077)
21 July 2020 - 27 July 2020	-0.164**	-0.103	-0.150
	(0.071)	(0.109)	(0.098)
28 July 2020 - 03 August 2020	-0.138	-0.085	-0.180
	(0.090)	(0.104)	(0.128)
04 August 2020 - 10 August 2020	-0.097	0.025	-0.081
	(0.059)	(0.093)	(0.079)
11 August 2020 - 17 August 2020	-0.062	-0.064	-0.078
	(0.063)	(0.090)	(0.091)
18 August 2020 - 24 August 2020	-0.197**	-0.167	-0.137
	(0.083)	(0.139)	(0.100)
25 August 2020 - 31 August 2020	-0.163**	-0.199*	-0.118
	(0.080)	(0.117)	(0.107)
01 September 2020 - 07 September 2020	-0.147**	-0.138	0.000
	(0.069)	(0.127)	(0.082)
08 September 2020 - 14 September 2020	-0.132*	-0.158*	-0.038
	(0.077)	(0.090)	(0.131)
15 September 2020 - 21 September 2020	-0.057	-0.056	0.045
	(0.093)	(0.123)	(0.121)

22 September 2020 - 28 September 2020	-0.097	-0.089	-0.086
	(0.063)	(0.088)	(0.093)
29 September 2020 - 05 October 2020	-0.058	-0.118	0.066
	(0.070)	(0.084)	(0.110)
06 October 2020 - 12 October 2020	-0.079	-0.177*	0.083
	(0.068)	(0.103)	(0.103)
13 October 2020 - 19 October 2020	-0.041	-0.137	0.103
	(0.070)	(0.084)	(0.139)
20 October 2020 - 26 October 2020	-0.098	-0.197*	0.102
	(0.074)	(0.108)	(0.114)
27 October 2020 - 02 November 2020	-0.059	-0.118	0.009
	(0.075)	(0.139)	(0.106)
03 November 2020 - 09 November 2020	-0.101	-0.113	-0.033
	(0.074)	(0.085)	(0.116)
10 November 2020 - 16 November 2020	-0.201***	-0.323***	0.030
	(0.077)	(0.088)	(0.107)
17 November 2020 - 23 November 2020	-0.186***	0.069	-0.150*
	(0.072)	(0.072)	(0.084)
24 November 2020 - 30 November 2020	-0.193**	-0.272**	-0.094
	(0.083)	(0.108)	(0.116)
Mean between 4 February - 16 March	0.610	0.631	0.567
Joint significance of all post-COVID weeks (p-value)	0.078	0.039	0.189
Observations	2,619	1,436	1,183
R^2	0.023	0.043	0.033
Household FE	YES	YES	YES

Table presents estimates from household fixed-effects regressions, with standard errors clustered at the household level.
* $P < 0.1$; ** $P < 0.05$; *** $P < 0.01$.

Supplementary Methods

Statistical analysis

In addition to linear probability regressions with household fixed-effects, clustering the standard errors at the household level, we also performed logit regressions and individual fixed-effects analysis, which yielded similar results. Tables containing these results can be found below.

Table A: Change in share of any health problems after 17 March, compared to the period 4 February - 16 March

	All members	Children	Adults
24 March 2020 - 30 March 2020	-0.167<br>(0.113)	-0.116<br>(0.136)	-0.288<br>(0.182)
31 March 2020 - 06 April 2020	-0.420***<br>(0.117)	-0.461***<br>(0.148)	-0.341*<br>(0.186)
07 April 2020 - 13 April 2020	-0.685***<br>(0.117)	-0.838***<br>(0.153)	-0.413**<br>(0.193)
14 April 2020 - 20 April 2020	-0.380***<br>(0.115)	-0.404***<br>(0.139)	-0.335*<br>(0.171)
21 April 2020 - 27 April 2020	-0.526***<br>(0.132)	-0.684***<br>(0.184)	-0.262<br>(0.179)
28 April 2020 - 04 May 2020	-0.442***<br>(0.115)	-0.605***<br>(0.147)	-0.174<br>(0.176)
05 May 2020 - 11 May 2020	0.208**<br>(0.091)	-0.061<br>(0.118)	0.626***<br>(0.132)
12 May 2020 - 18 May 2020	-0.238**<br>(0.103)	-0.767***<br>(0.157)	0.334**<br>(0.140)
19 May 2020 - 25 May 2020	-0.316***<br>(0.103)	-0.701***<br>(0.153)	0.154<br>(0.144)
26 May 2020 - 01 June 2020	-0.406***<br>(0.111)	-0.650***<br>(0.155)	-0.076<br>(0.147)
16 June 2020 - 22 June 2020	0.012<br>(0.101)	-0.323**<br>(0.146)	0.424***<br>(0.130)
23 June 2020 - 29 June 2020	0.059<br>(0.108)	-0.345**<br>(0.156)	0.532***<br>(0.138)
30 June 2020 - 06 July 2020	-0.001<br>(0.101)	-0.263*<br>(0.135)	0.353**<br>(0.139)
07 July 2020 - 13 July 2020	0.019<br>(0.112)	-0.182<br>(0.152)	0.308**<br>(0.143)
14 July 2020 - 20 July 2020	0.173*<br>(0.092)	0.002<br>(0.125)	0.430***<br>(0.131)
21 July 2020 - 27 July 2020	-0.122<br>(0.108)	-0.315**<br>(0.145)	0.141<br>(0.139)
28 July 2020 - 03 August 2020	-0.287**<br>(0.125)	-0.491***<br>(0.160)	0.023<br>(0.146)
04 August 2020 - 10 August 2020	-0.277**<br>(0.111)	-0.975***<br>(0.180)	0.360**<br>(0.146)
11 August 2020 - 17 August 2020	-0.072<br>(0.100)	-0.165<br>(0.118)	0.089<br>(0.153)

18 August 2020 - 24 August 2020	-0.427***	-1.296***	0.287**
	(0.114)	(0.210)	(0.138)
25 August 2020 - 31 August 2020	-0.496***	-0.715***	-0.166
	(0.121)	(0.160)	(0.150)
01 September 2020 - 07 September 2020	-0.572***	-1.563***	0.192
	(0.126)	(0.214)	(0.161)
08 September 2020 - 14 September 2020	-0.559***	-0.599***	-0.484***
	(0.111)	(0.148)	(0.176)
15 September 2020 - 21 September 2020	-0.519***	-0.678***	-0.262
	(0.126)	(0.165)	(0.161)
22 September 2020 - 28 September 2020	-0.418***	-0.591***	-0.141
	(0.105)	(0.133)	(0.155)
29 September 2020 - 05 October 2020	-0.559***	-0.774***	-0.233
	(0.129)	(0.174)	(0.155)
06 October 2020 - 12 October 2020	-0.289***	-0.486***	0.021
	(0.106)	(0.136)	(0.145)
13 October 2020 - 19 October 2020	-0.299***	-0.335**	-0.229
	(0.107)	(0.130)	(0.162)
20 October 2020 - 26 October 2020	-0.516***	-0.636***	-0.310*
	(0.116)	(0.152)	(0.166)
27 October 2020 - 02 November 2020	-0.554***	-1.765***	0.281**
	(0.117)	(0.235)	(0.143)
03 November 2020 - 09 November 2020	-0.294**	-0.508***	0.035
	(0.115)	(0.144)	(0.149)
10 November 2020 - 16 November 2020	-0.082	-0.172	0.076
	(0.115)	(0.141)	(0.142)
17 November 2020 - 23 November 2020	-0.786***	-2.010***	0.020
	(0.128)	(0.286)	(0.154)
24 November 2020 - 30 November 2020	-0.287**	-0.412***	-0.074
	(0.120)	(0.154)	(0.163)
Joint significance of all post-COVID weeks (p-value)	0.000	0.000	0.000
Observations	55,923	34,719	21,204
Pseudo-R2	0.008	0.020	0.010
Household FE	NO	NO	NO

Table presents estimates from logit regressions, with standard errors clustered at the household level. * $P < 0.1$; ** $P < 0.05$; *** $P < 0.01$.

Table B: Change in share of Gastrointestinal after 17 March, compared to the period 4 February - 16 March

	All members	Children	Adults
24 March 2020 - 30 March 2020	-0.469	-0.421	-0.612
	(0.371)	(0.434)	(0.711)
31 March 2020 - 06 April 2020	-0.713	-0.363	
	(0.517)	(0.539)	
07 April 2020 - 13 April 2020	-0.673*	-1.194**	0.030
	(0.399)	(0.602)	(0.530)
14 April 2020 - 20 April 2020	-0.495	-0.731	-0.092
	(0.353)	(0.470)	(0.522)

21 April 2020 - 27 April 2020	-0.610	-0.947*	-0.098
	(0.375)	(0.516)	(0.548)
28 April 2020 - 04 May 2020	-0.461	-0.343	-0.783
	(0.342)	(0.398)	(0.740)
05 May 2020 - 11 May 2020	0.149	0.035	0.375
	(0.281)	(0.341)	(0.447)
12 May 2020 - 18 May 2020	0.387	0.210	0.684*
	(0.242)	(0.323)	(0.370)
19 May 2020 - 25 May 2020	-0.298	-0.417	-0.073
	(0.334)	(0.449)	(0.497)
26 May 2020 - 01 June 2020	0.165	-0.691	0.930**
	(0.340)	(0.613)	(0.374)
16 June 2020 - 22 June 2020	0.266	-0.090	0.733**
	(0.249)	(0.360)	(0.339)
23 June 2020 - 29 June 2020	0.393	0.055	0.845**
	(0.255)	(0.410)	(0.348)
30 June 2020 - 06 July 2020	0.305	0.351	0.246
	(0.234)	(0.287)	(0.420)
07 July 2020 - 13 July 2020	0.023	-0.020	0.132
	(0.261)	(0.352)	(0.415)
14 July 2020 - 20 July 2020	0.030	-0.123	0.300
	(0.275)	(0.367)	(0.424)
21 July 2020 - 27 July 2020	0.066	-0.108	0.351
	(0.318)	(0.425)	(0.416)
28 July 2020 - 03 August 2020	-0.373	-0.771	0.168
	(0.396)	(0.555)	(0.462)
04 August 2020 - 10 August 2020	0.232	-0.274	0.797*
	(0.299)	(0.359)	(0.434)
11 August 2020 - 17 August 2020	-0.013	0.109	-0.315
	(0.293)	(0.351)	(0.546)
18 August 2020 - 24 August 2020	0.048	-0.781*	0.775**
	(0.277)	(0.452)	(0.367)
25 August 2020 - 31 August 2020	-0.538	-0.849*	-0.069
	(0.328)	(0.461)	(0.479)
01 September 2020 - 07 September 2020	0.243	-0.703	1.024**
	(0.374)	(0.479)	(0.445)
08 September 2020 - 14 September 2020	-0.378	-0.194	-0.927
	(0.350)	(0.373)	(0.741)
15 September 2020 - 21 September 2020	-0.235	-0.342	-0.025
	(0.359)	(0.380)	(0.574)
22 September 2020 - 28 September 2020	-0.032	-0.027	-0.029
	(0.308)	(0.397)	(0.441)
29 September 2020 - 05 October 2020	-0.492	-0.807	-0.018
	(0.512)	(0.845)	(0.497)
06 October 2020 - 12 October 2020	0.094	0.064	0.166
	(0.294)	(0.340)	(0.448)
13 October 2020 - 19 October 2020	0.248	0.285	0.170
	(0.258)	(0.309)	(0.447)
20 October 2020 - 26 October 2020	-0.158	-0.229	-0.007
	(0.312)	(0.395)	(0.498)
27 October 2020 - 02 November 2020	-0.214	-0.251	-0.110
	(0.299)	(0.377)	(0.477)

03 November 2020 - 09 November 2020	-0.996**	-1.313**	-0.520
	(0.422)	(0.602)	(0.594)
10 November 2020 - 16 November 2020	-0.200	-0.998*	0.596
	(0.285)	(0.516)	(0.371)
17 November 2020 - 23 November 2020	-0.362	-0.916*	0.231
	(0.324)	(0.518)	(0.420)
24 November 2020 - 30 November 2020	-0.336	-0.976*	0.390
	(0.352)	(0.525)	(0.421)
Joint significance of all post-COVID weeks (p-value)	0.001	0.025	0.000
Observations	55,923	34,719	20,749
Pseudo-R2	0.009	0.014	0.017
Household FE	NO	NO	NO

Table presents estimates from logit regressions, with standard errors clustered at the household level. * $P < 0.1$; ** $P < 0.05$; *** $P < 0.01$.

Table C: Change in share of Malaria after 17 March, compared to the period 4 February - 16 March

	All members	Children	Adults
24 March 2020 - 30 March 2020	-0.222	-0.153	-0.654
	(0.146)	(0.161)	(0.402)
31 March 2020 - 06 April 2020	-0.452***	-0.465**	-0.424
	(0.172)	(0.181)	(0.332)
07 April 2020 - 13 April 2020	-0.663***	-0.806***	-0.250
	(0.168)	(0.195)	(0.343)
14 April 2020 - 20 April 2020	-0.187	-0.266	0.075
	(0.146)	(0.162)	(0.277)
21 April 2020 - 27 April 2020	-0.425***	-0.653***	0.184
	(0.162)	(0.196)	(0.265)
28 April 2020 - 04 May 2020	-0.413***	-0.582***	0.082
	(0.156)	(0.180)	(0.275)
05 May 2020 - 11 May 2020	-0.152	-0.238	0.134
	(0.143)	(0.162)	(0.275)
12 May 2020 - 18 May 2020	-0.639***	-1.620***	0.581***
	(0.166)	(0.291)	(0.206)
19 May 2020 - 25 May 2020	-0.432***	-0.781***	0.344
	(0.153)	(0.223)	(0.211)
26 May 2020 - 01 June 2020	-0.833***	-0.928***	-0.459
	(0.213)	(0.257)	(0.313)
16 June 2020 - 22 June 2020	-0.104	-0.420**	0.626***
	(0.135)	(0.178)	(0.212)
23 June 2020 - 29 June 2020	-0.295*	-0.671***	0.503**
	(0.154)	(0.198)	(0.217)
30 June 2020 - 06 July 2020	-0.374***	-0.452***	-0.042
	(0.144)	(0.173)	(0.272)
07 July 2020 - 13 July 2020	-0.473***	-0.711***	0.152
	(0.149)	(0.190)	(0.254)
14 July 2020 - 20 July 2020	-0.260*	-0.406**	0.211
	(0.146)	(0.182)	(0.252)
21 July 2020 - 27 July 2020	-0.723***	-0.723***	-0.544
	(0.173)	(0.201)	(0.333)
28 July 2020 - 03 August 2020	-0.723***	-0.797***	-0.436
	(0.178)	(0.207)	(0.325)
04 August 2020 - 10 August 2020	-0.870***	-2.186***	0.420*
	(0.173)	(0.357)	(0.228)
11 August 2020 - 17 August 2020	-0.555***	-0.677***	-0.154
	(0.149)	(0.176)	(0.278)
18 August 2020 - 24 August 2020	-1.160***	-2.101***	0.007
	(0.219)	(0.405)	(0.267)
25 August 2020 - 31 August 2020	-0.917***	-1.173***	-0.261
	(0.170)	(0.219)	(0.271)
01 September 2020 - 07 September 2020	-1.104***	-2.141***	0.111
	(0.181)	(0.354)	(0.247)
08 September 2020 - 14 September 2020	-0.752***	-0.748***	-0.718**
	(0.158)	(0.187)	(0.339)
15 September 2020 - 21 September 2020	-1.050***	-0.943***	-1.558***
	(0.220)	(0.234)	(0.510)

22 September 2020 - 28 September 2020	-0.761***	-0.817***	-0.537
	(0.148)	(0.176)	(0.330)
29 September 2020 - 05 October 2020	-1.285***	-1.544***	-0.623*
	(0.201)	(0.250)	(0.353)
06 October 2020 - 12 October 2020	-0.778***	-0.814***	-0.623*
	(0.169)	(0.186)	(0.339)
13 October 2020 - 19 October 2020	-0.756***	-0.788***	-0.620*
	(0.178)	(0.188)	(0.321)
20 October 2020 - 26 October 2020	-0.752***	-0.914***	-0.268
	(0.165)	(0.206)	(0.289)
27 October 2020 - 02 November 2020	-0.967***	-2.453***	0.380*
	(0.192)	(0.464)	(0.227)
03 November 2020 - 09 November 2020	-0.585***	-0.733***	-0.129
	(0.174)	(0.193)	(0.278)
10 November 2020 - 16 November 2020	-0.672***	-0.648***	-0.712**
	(0.164)	(0.167)	(0.357)
17 November 2020 - 23 November 2020	-1.291***	-2.604***	-0.005
	(0.231)	(0.453)	(0.260)
24 November 2020 - 30 November 2020	-0.783***	-0.809***	-0.662*
	(0.179)	(0.209)	(0.344)
Joint significance of all post-COVID weeks (p-value)	0.000	0.000	0.000
Observations	55,923	34,719	21,204
Pseudo-R2	0.015	0.032	0.018
Household FE	NO	NO	NO

Table presents estimates from logit regressions, with standard errors clustered at the household level. * $P < 0.1$; ** $P < 0.05$; *** $P < 0.01$.

Table D: Change in share of NCD after 17 March, compared to the period 4 February - 16 March

	All members	Children	Adults
24 March 2020 - 30 March 2020	-0.329	-0.182	-0.441
	(0.248)	(0.391)	(0.328)
31 March 2020 - 06 April 2020	0.102	0.318	-0.092
	(0.229)	(0.363)	(0.265)
07 April 2020 - 13 April 2020	-0.021	0.007	-0.014
	(0.207)	(0.332)	(0.264)
14 April 2020 - 20 April 2020	0.207	0.445	-0.016
	(0.182)	(0.275)	(0.243)
21 April 2020 - 27 April 2020	0.183	0.351	0.033
	(0.194)	(0.285)	(0.276)
28 April 2020 - 04 May 2020	0.412**	0.445	0.376*
	(0.178)	(0.272)	(0.221)
05 May 2020 - 11 May 2020	0.804***	0.514*	0.998***
	(0.160)	(0.269)	(0.188)
12 May 2020 - 18 May 2020	0.614***	0.160	0.772***
	(0.167)	(0.321)	(0.186)
19 May 2020 - 25 May 2020	0.558***	0.468	0.555**
	(0.188)	(0.305)	(0.222)
26 May 2020 - 01 June 2020	0.540***	0.580**	0.440**
	(0.177)	(0.284)	(0.220)
16 June 2020 - 22 June 2020	0.922***	0.632**	1.004***
	(0.153)	(0.271)	(0.176)
23 June 2020 - 29 June 2020	0.937***	0.837***	0.921***
	(0.175)	(0.271)	(0.205)
30 June 2020 - 06 July 2020	0.849***	0.595**	0.934***
	(0.158)	(0.247)	(0.197)
07 July 2020 - 13 July 2020	0.705***	0.594**	0.710***
	(0.164)	(0.280)	(0.207)
14 July 2020 - 20 July 2020	1.105***	1.102***	1.052***
	(0.155)	(0.255)	(0.194)
21 July 2020 - 27 July 2020	0.732***	0.943***	0.473**
	(0.156)	(0.248)	(0.195)
28 July 2020 - 03 August 2020	0.201	0.126	0.213
	(0.187)	(0.300)	(0.233)
04 August 2020 - 10 August 2020	0.486***	0.212	0.532***
	(0.178)	(0.305)	(0.199)
11 August 2020 - 17 August 2020	0.572***	0.577**	0.537***
	(0.168)	(0.264)	(0.201)
18 August 2020 - 24 August 2020	0.509***	-0.176	0.700***
	(0.158)	(0.327)	(0.179)
25 August 2020 - 31 August 2020	0.517***	0.620**	0.409**
	(0.177)	(0.287)	(0.204)
01 September 2020 - 07 September 2020	0.454**	0.196	0.507***
	(0.180)	(0.320)	(0.196)
08 September 2020 - 14 September 2020	0.355*	0.526*	0.183
	(0.184)	(0.288)	(0.242)
15 September 2020 - 21 September 2020	0.241	0.017	0.354
	(0.205)	(0.425)	(0.225)
22 September 2020 - 28 September 2020	0.280	0.137	0.350

	(0.199)	(0.318)	(0.236)
29 September 2020 - 05 October 2020	0.225	-0.046	0.362
	(0.178)	(0.330)	(0.223)
06 October 2020 - 12 October 2020	0.539***	0.198	0.721***
	(0.155)	(0.285)	(0.194)
13 October 2020 - 19 October 2020	0.408**	0.350	0.434**
	(0.181)	(0.282)	(0.219)
20 October 2020 - 26 October 2020	0.123	0.076	0.139
	(0.210)	(0.319)	(0.244)
27 October 2020 - 02 November 2020	0.263	-0.529	0.487**
	(0.195)	(0.429)	(0.222)
03 November 2020 - 09 November 2020	0.503***	0.468	0.508**
	(0.182)	(0.299)	(0.212)
10 November 2020 - 16 November 2020	0.540***	0.734***	0.345
	(0.182)	(0.268)	(0.232)
17 November 2020 - 23 November 2020	0.284	-0.786*	0.545**
	(0.195)	(0.424)	(0.225)
24 November 2020 - 30 November 2020	0.574***	0.562*	0.566**
	(0.196)	(0.312)	(0.228)
Joint significance of all post-COVID weeks (p-value)	0.000	0.000	0.000
Observations	55,923	34,719	21,204
Pseudo-R2	0.011	0.013	0.013
Household FE	NO	NO	NO

Table presents estimates from logit regressions, with standard errors clustered at the household level. * $P < 0.1$; ** $P < 0.05$; *** $P < 0.01$.

Table E: Change in share of Respiratory/air-borne after 17 March, compared to the period 4 February - 16 March

	All members	Children	Adults
24 March 2020 - 30 March 2020	0.150	0.154	0.092
	(0.153)	(0.173)	(0.325)
31 March 2020 - 06 April 2020	-0.231	-0.439*	0.343
	(0.192)	(0.230)	(0.321)
07 April 2020 - 13 April 2020	-0.992***	-1.069***	-0.769
	(0.228)	(0.267)	(0.471)
14 April 2020 - 20 April 2020	-0.482**	-0.684***	0.082
	(0.211)	(0.243)	(0.314)
21 April 2020 - 27 April 2020	-0.748***	-0.895***	-0.301
	(0.261)	(0.335)	(0.412)
28 April 2020 - 04 May 2020	-0.903***	-1.344***	0.006
	(0.233)	(0.350)	(0.328)
05 May 2020 - 11 May 2020	0.115	-0.091	0.687***
	(0.160)	(0.192)	(0.256)
12 May 2020 - 18 May 2020	-0.707***	-1.260***	0.263
	(0.214)	(0.314)	(0.304)
19 May 2020 - 25 May 2020	-0.880***	-1.343***	0.019
	(0.202)	(0.287)	(0.284)
26 May 2020 - 01 June 2020	-1.257***	-1.352***	-0.879*
	(0.293)	(0.323)	(0.508)
16 June 2020 - 22 June 2020	-0.428**	-0.634**	0.155
	(0.207)	(0.247)	(0.320)
23 June 2020 - 29 June 2020	-0.125	-0.581*	0.754***
	(0.205)	(0.302)	(0.285)
30 June 2020 - 06 July 2020	-0.142	-0.376*	0.484*
	(0.184)	(0.227)	(0.292)
07 July 2020 - 13 July 2020	0.120	0.011	0.527*
	(0.211)	(0.242)	(0.283)
14 July 2020 - 20 July 2020	0.210	0.096	0.623**
	(0.153)	(0.181)	(0.256)
21 July 2020 - 27 July 2020	-0.372*	-0.779***	0.453
	(0.222)	(0.285)	(0.290)
28 July 2020 - 03 August 2020	0.043	-0.279	0.799***
	(0.191)	(0.238)	(0.237)
04 August 2020 - 10 August 2020	-0.399*	-0.971***	0.564**
	(0.208)	(0.317)	(0.284)
11 August 2020 - 17 August 2020	0.100	-0.012	0.487*
	(0.160)	(0.180)	(0.278)
18 August 2020 - 24 August 2020	-0.616***	-1.525***	0.541*
	(0.217)	(0.394)	(0.281)
25 August 2020 - 31 August 2020	-0.583**	-0.805***	0.023
	(0.227)	(0.257)	(0.322)
01 September 2020 - 07 September 2020	-1.398***	-3.249***	0.000
	(0.384)	(0.715)	(0.435)
08 September 2020 - 14 September 2020	-0.569***	-0.628***	-0.327
	(0.192)	(0.223)	(0.312)
15 September 2020 - 21 September 2020	-0.381*	-0.493**	-0.003
	(0.210)	(0.227)	(0.325)

22 September 2020 - 28 September 2020	-0.477**	-0.739***	0.193
	(0.186)	(0.200)	(0.315)
29 September 2020 - 05 October 2020	-0.332	-0.581**	0.319
	(0.227)	(0.271)	(0.284)
06 October 2020 - 12 October 2020	-0.203	-0.355*	0.263
	(0.191)	(0.216)	(0.294)
13 October 2020 - 19 October 2020	-0.187	-0.211	-0.068
	(0.184)	(0.195)	(0.307)
20 October 2020 - 26 October 2020	-0.423**	-0.500**	-0.142
	(0.200)	(0.222)	(0.355)
27 October 2020 - 02 November 2020	-0.600***	-2.352***	0.801***
	(0.230)	(0.462)	(0.280)
03 November 2020 - 09 November 2020	0.029	-0.233	0.699***
	(0.172)	(0.192)	(0.255)
10 November 2020 - 16 November 2020	0.337*	0.233	0.705***
	(0.183)	(0.195)	(0.266)
17 November 2020 - 23 November 2020	-0.856***	-2.543***	0.511*
	(0.239)	(0.617)	(0.282)
24 November 2020 - 30 November 2020	-0.012	-0.089	0.274
	(0.171)	(0.199)	(0.293)
Joint significance of all post-COVID weeks (p-value)	0.000	0.000	0.000
Observations	55,923	34,719	21,204
Pseudo-R2	0.015	0.033	0.014
Household FE	NO	NO	NO

Table presents estimates from logit regressions, with standard errors clustered at the household level. * $P < 0.1$; ** $P < 0.05$; *** $P < 0.01$.

Table F: Change in share of Consulted formal provider for health problem after 17 March, compared to the period 4 February - 16 March

	All members	Children	Adults
24 March 2020 - 30 March 2020	0.138	0.188	-0.048
	(0.314)	(0.377)	(0.487)
31 March 2020 - 06 April 2020	-0.215	-0.148	-0.559
	(0.296)	(0.385)	(0.550)
07 April 2020 - 13 April 2020	0.455	0.381	0.604
	(0.344)	(0.442)	(0.571)
14 April 2020 - 20 April 2020	-0.388	-0.361	-0.438
	(0.284)	(0.357)	(0.425)
21 April 2020 - 27 April 2020	-0.189	-0.248	-0.070
	(0.335)	(0.417)	(0.504)
28 April 2020 - 04 May 2020	-0.956***	-1.106***	-0.706
	(0.296)	(0.412)	(0.433)
05 May 2020 - 11 May 2020	-0.089	-0.039	-0.077
	(0.327)	(0.409)	(0.404)
12 May 2020 - 18 May 2020	-0.040	0.745	-0.325
	(0.314)	(0.587)	(0.399)
19 May 2020 - 25 May 2020	-0.281	-0.130	-0.271
	(0.297)	(0.473)	(0.381)
26 May 2020 - 01 June 2020	-0.238	-0.130	-0.271
	(0.378)	(0.468)	(0.475)
16 June 2020 - 22 June 2020	-0.239	-0.425	0.017
	(0.285)	(0.411)	(0.361)
23 June 2020 - 29 June 2020	-0.558**	-0.264	-0.661*
	(0.263)	(0.406)	(0.353)
30 June 2020 - 06 July 2020	-0.177	0.093	-0.362
	(0.268)	(0.370)	(0.341)
07 July 2020 - 13 July 2020	-0.393	-0.313	-0.371
	(0.266)	(0.384)	(0.332)
14 July 2020 - 20 July 2020	-0.715***	-0.613*	-0.770**
	(0.257)	(0.344)	(0.347)
21 July 2020 - 27 July 2020	-0.416	-0.248	-0.508
	(0.284)	(0.420)	(0.369)
28 July 2020 - 03 August 2020	-0.376	0.077	-1.187**
	(0.361)	(0.435)	(0.512)
04 August 2020 - 10 August 2020	-0.202	0.494	-0.314
	(0.286)	(0.618)	(0.352)
11 August 2020 - 17 August 2020	-0.360	-0.366	-0.328
	(0.265)	(0.332)	(0.386)
18 August 2020 - 24 August 2020	-0.701**	-0.787	-0.529
	(0.325)	(0.538)	(0.390)
25 August 2020 - 31 August 2020	-0.668**	-0.616	-0.619
	(0.316)	(0.448)	(0.405)
01 September 2020 - 07 September 2020	-0.519*	-0.248	-0.472
	(0.311)	(0.532)	(0.402)
08 September 2020 - 14 September 2020	-0.653**	-0.718*	-0.559
	(0.310)	(0.374)	(0.565)
15 September 2020 - 21 September 2020	-0.486	-0.456	-0.438
	(0.340)	(0.484)	(0.457)

22 September 2020 - 28 September 2020	-0.268	-0.150	-0.425
	(0.287)	(0.367)	(0.421)
29 September 2020 - 05 October 2020	-0.606*	-0.623	-0.494
	(0.356)	(0.484)	(0.414)
06 October 2020 - 12 October 2020	-0.512*	-0.772**	-0.117
	(0.283)	(0.385)	(0.404)
13 October 2020 - 19 October 2020	-0.064	-0.113	0.017
	(0.285)	(0.329)	(0.491)
20 October 2020 - 26 October 2020	-0.787**	-0.963**	-0.494
	(0.313)	(0.398)	(0.437)
27 October 2020 - 02 November 2020	-0.519	-0.536	-0.371
	(0.330)	(0.565)	(0.390)
03 November 2020 - 09 November 2020	-0.512	-0.600	-0.340
	(0.336)	(0.425)	(0.431)
10 November 2020 - 16 November 2020	-0.710**	-1.013**	-0.202
	(0.323)	(0.404)	(0.395)
17 November 2020 - 23 November 2020	-0.831**	1.544	-1.252***
	(0.362)	(1.077)	(0.440)
24 November 2020 - 30 November 2020	-0.753**	-0.772*	-0.677
	(0.339)	(0.411)	(0.438)
Joint significance of all post-COVID weeks (p-value)	0.087	0.087	0.530
Observations	2,619	1,436	1,183
Pseudo-R2	0.016	0.028	0.017
Household FE	NO	NO	NO

Table presents estimates from logit regressions, with standard errors clustered at the household level. * $P < 0.1$; ** $P < 0.05$; *** $P < 0.01$.

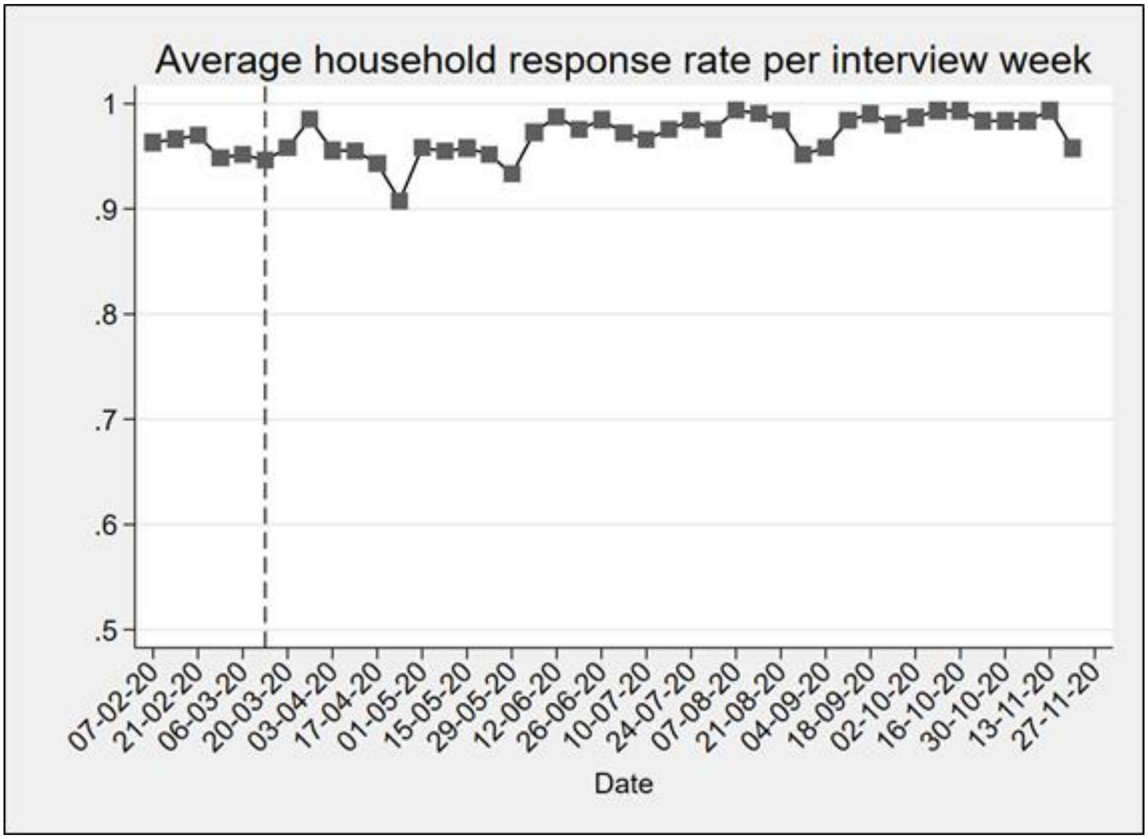

Supplementary Figure 1. Response rate to the weekly diaries interviews during the 40-week study period. The black squares show the response rate to weekly diaries interviews, the vertical dashed line shows the time cut-off for before and during COVID-19 comparisons. Between end of March and the first week of April 2020, mobile phones were provided to the households to continue the weekly interviews via telephone calls.

A

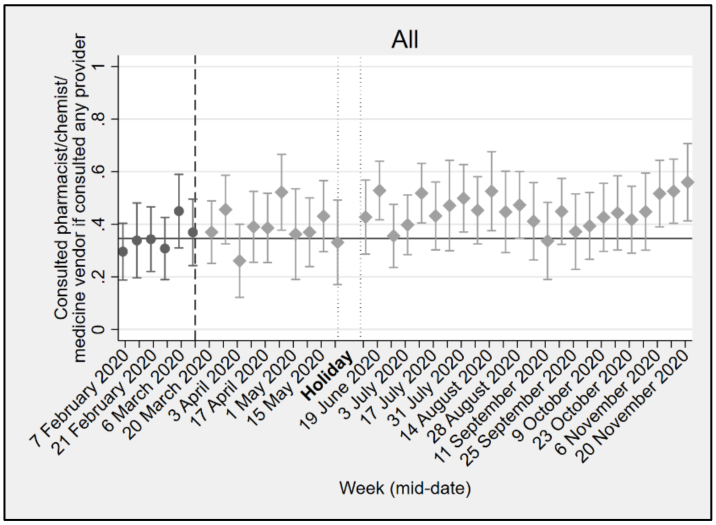

B

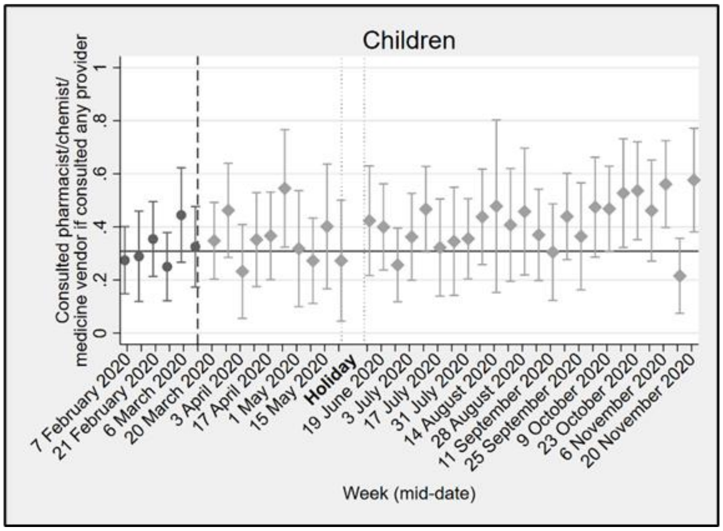

C

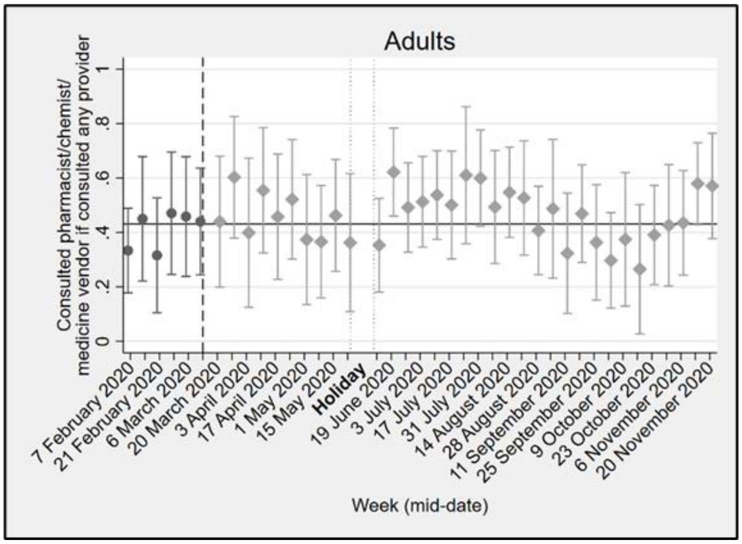

Supplementary Figure 2. Consultations at pharmacists, chemists, or medicine vendors conditional on seeking care.

These figures show the weekly average number of times that individuals living in these households consulted a pharmacist, chemist, or medicine vendor conditional on seeking care from February-November 2020. A, all household members; B, Children (< 18 years old); C, Adults (≥ 18 years old). The vertical dashed line shows the time cut-off for before and during COVID-19 comparisons; the two dotted vertical lines show the holiday period without interviews; and the horizontal line shows the average of consultations before COVID-19. Error bars show 95% confidence intervals.
